# Novel Quinoline Compounds Active in Cancer Cells through Coupled DNA Methyltransferase Inhibition and Degradation

**DOI:** 10.3390/cancers12020447

**Published:** 2020-02-14

**Authors:** Clemens Zwergel, Rossella Fioravanti, Giulia Stazi, Federica Sarno, Cecilia Battistelli, Annalisa Romanelli, Angela Nebbioso, Eduarda Mendes, Alexandra Paulo, Raffaele Strippoli, Marco Tripodi, Dany Pechalrieu, Paola B. Arimondo, Teresa De Luca, Donatella Del Bufalo, Daniela Trisciuoglio, Lucia Altucci, Sergio Valente, Antonello Mai

**Affiliations:** 1 Department of Drug Chemistry and Technologies, Sapienza University of Rome, P. le A. Moro 5, 00185 Rome, Italy; clemens.zwergel@uniroma1.it (C.Z.); rossella.fioravanti@uniroma1.it (R.F.); giulia.stazi@uniroma1.it (G.S.); annalisa.romanelli@uniroma1.it (A.R.); 2 Department of Precision Medicine, University of Studi della Campania Luigi Vanvitelli, Vico L. De Crecchio 7, 80138 Naples, Italy; federica.sarno@unicampania.it (F.S.); angela.nebbioso@unicampania.it (A.N.); lucia.altucci@unicampania.it (L.A.); 3 Department of Molecular Medicine, Sapienza University of Rome, Viale Regina Elena 324, 00161 Rome, Italy; cecilia.battistelli@uniroma1.it (C.B.); raffaele.strippoli@uniroma1.it (R.S.); marco.tripodi@uniroma1.it (M.T.); 4 Research Institute for Medicines, Medicinal Chemistry Group, Faculty of Pharmacy, Universidade de Lisboa, 1649 003 Lisbon, Portugal; ermendes@ff.ulisboa.pt (E.M.); mapaulo@ff.ulisboa.pt (A.P.); 5 National Institute for Infectious Diseases L. Spallanzani, IRCCS, Via Portuense, 292, 00149 Rome, Italy; 6 Istituto Pasteur- Fondazione Cenci Bolognetti, Department of Molecular Medicine, Sapienza Università di Roma, 00185 Rome, Italy; 7 ETaC CNRS FRE3600, LMBE, 118 route de Narbonne, 31062 Toulouse, France; dany.pechalrieu@gmail.com (D.P.); paola.arimondo@cnrs.fr (P.B.A.); 8 Epigenetic Chemical Biology, Institute Pasteur, CNRS UMR3523, 28 rue du Docteur Roux, 75724 Paris, France; 9 Preclinical Models and New Therapeutic Agents Unit, IRCCS-Regina Elena National Cancer Institute, Via Elio Chianesi 53, 00144 Rome, Italy; teresa.deluca@ifo.gov.it (T.D.L.); donatella.delbufalo@ifo.gov.it (D.D.B.); 10 Institute of Molecular Biology and Pathology, National Research Council (CNR), Via Degli Apuli 4, 00185 Rome, Italy

**Keywords:** drug discovery, medicinal chemistry, DNA methyltransferase, enzyme inhibition, protein degradation, apoptosis

## Abstract

DNA methyltransferases (DNMTs) play a relevant role in epigenetic control of cancer cell survival and proliferation. Since only two DNMT inhibitors (azacitidine and decitabine) have been approved to date for the treatment of hematological malignancies, the development of novel potent and specific inhibitors is urgent. Here we describe the design, synthesis, and biological evaluation of a new series of compounds acting at the same time as DNMTs (mainly DNMT3A) inhibitors and degraders. Tested against leukemic and solid cancer cell lines, 2a–c and 4a–c (the last only for leukemias) displayed up to submicromolar antiproliferative activities. In HCT116 cells, such compounds induced EGFP gene expression in a promoter demethylation assay, confirming their demethylating activity in cells. In the same cell line, 2b and 4c chosen as representative samples induced DNMT1 and -3A protein degradation, suggesting for these compounds a double mechanism of DNMT3A inhibition and DNMT protein degradation.

## 1. Introduction

DNA methylation is one of the most extensively studied epigenetic marks, playing an important role in genomic imprinting; DNA repair; X-chromosome inactivation; and silencing of retrotransposons, repetitive elements, and tissue-specific genes. Both the hypo- and hypermethylation of different genome regions play a crucial role in tumorigenesis [1]. For this reason, focal hypermethylation has been intensively studied in cancer, as a causative factor of transcriptional inactivation of tumor suppressor genes (TSG) [2]. Some TSGs, including GSTP1, MGMT, CDH1, P16, RAR-β2, septin 9 (SEPT9), syndecan 2 (SDC2), cyclin-dependent kinase inhibitor 2A (CDKN2A), and short stature homeobox 2 (SHOX2), have been validated to be silenced through DNA methylation in various types of cancer [3]. DNA methylation occurs mainly in the context of CpG dinucleotides clustered in CpG islands [4,5,6] and is governed by catalytically active DNA methyltransferase (DNMT) enzymes, which methylate the 5-position of cytosine by using *S*-adenosyl-l-methionine (SAM) as the cosubstrate. DNMT1 is responsible for DNA methylation by preserving the DNA methylation pattern during the replication; DNMT3A and DNMT3B perform de novo DNA methylation either on hemi methylated or unmethylated double stranded DNA; DNMT2 catalyzes the methylation on tRNA, and is called TRDMT1; finally, DNMT3-Like protein (DNMT3L) does not display catalytic activity but works as a regulatory factor in complex with DNMT3A and -B [7,8]. Like for other epigenetic modifications, the reversibility of DNA methylation represents an interesting therapeutic strategy, especially in cancer [9,10,11]. Hence, several studies clearly showed that the DNMTs’ depletion by RNA interference is sufficient to provide TSGs re-expression and cell growth arrest and to promote re-differentiation of several cancer cell types including lung, esophagus, stomach, breast, cervix, brain, and head and neck cancer [10,11,12]. 

**Figure 1:**
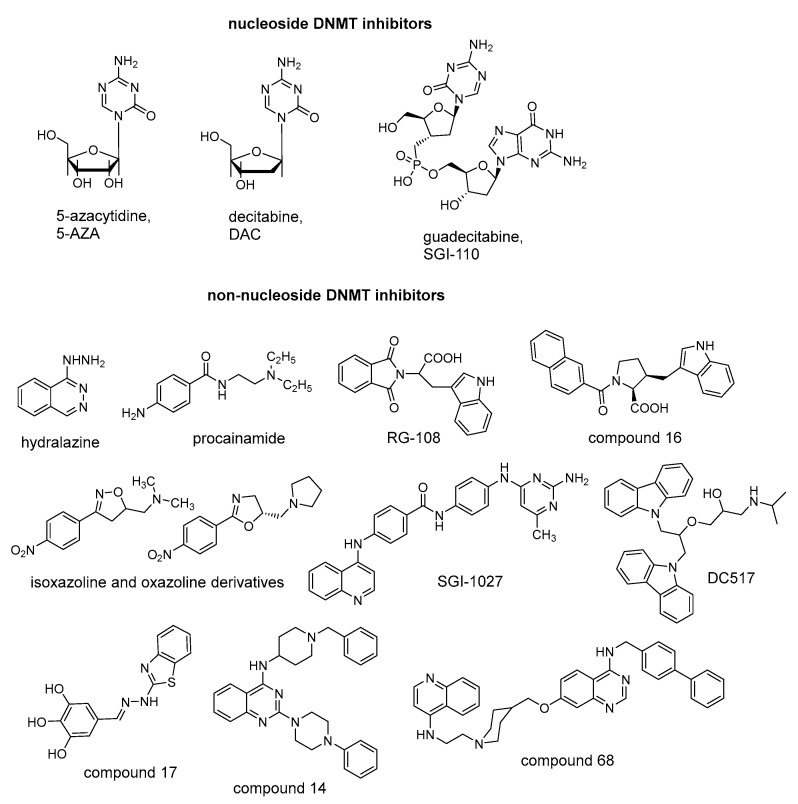
Known nucleoside and non-nucleoside DNMTs inhibitors.

Two families of DNMT inhibitors (DNMTi) have been described so far as useful tools to reactivate TSGs and to reprogram cancer cells towards growth arrest and death: the covalent, irreversible nucleoside inhibitors and the non-nucleoside inhibitors [12,13]. Two nucleoside-like DNMTi (5-azacytidine, 5-AZA, and 5-aza-2′-deoxycytidine, DAC, Figure 1) have been approved by the FDA for clinical use against myelodysplastic syndromes and acute myeloid leukemia, but despite their high efficacy, their use is limited by poor bioavailability, chemical instability, and severe side-effects [13]. Fewer limitations are shown by guadecitabine [14] (Figure 1), a dinucleotide derivative of DAC with better pharmacokinetic properties, currently under phase I/II clinical trials for the treatment of either hematological or solid cancers, alone or in combination with other chemotherapeutic agents [15]. Various natural and synthetic non-nucleoside inhibitors have been reported recently, but even though they are less toxic than azanucleosides, they share low potency and/or low specificity for DNMTs. Some natural compounds such as mahanine and laccaic acid A have been recently reviewed as DNMTi capable of reactivating several TSGs [8,16]. Repositioning of old drugs (i.e., hydralazine, in phase II clinical trials against solid cancers [3], and procainamide, Figure 1) [16] or identification of new chemical entities such as RG-108 [17] and its naphthoylproline analog [18], isoxazoline [19] and oxazoline [20] derivatives, and SGI-1027 [21] (Figure 1) provided the structural bases for developing novel and more potent DNMTi. Furthermore, carbazoles (DC517) [22], hydrazineylidenemethyl benzothiazoles (compound 17) [23], and some DNMT3A-selective quinazolines (compound 14 [24] and compound 68 [25]) (Figure 1) enriched the recent literature on DNMTi, but still need further studies for clinical development.

**Figure 2:**
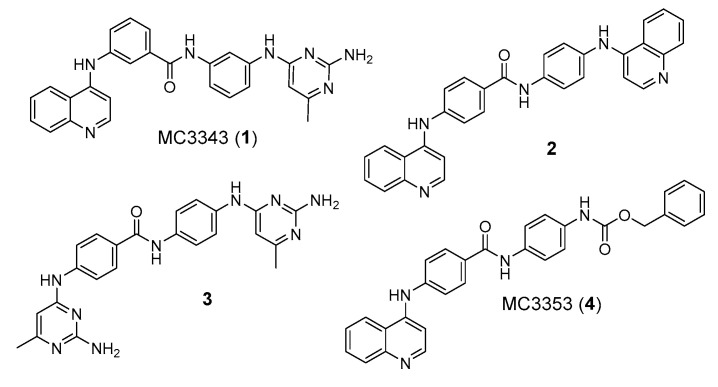
Regioisomers and analogues of SGI-1027 described by our research group.

In 2014, through a process of hit-to-lead optimization, we identified the *meta*-*meta* isomer of SGI-1027, **1** (MC3343, Figure 2), as a non-nucleoside DNMTi more potent and selective than SGI-1027 toward other SAM-dependent methyltransferases [26,27,28]. Compound **1** displayed single-digit micromolar potency against a panel of cancer cells including mouse medulloblastoma stem cells, showing less toxicity than SGI-1027 in peripheral blood mononuclear cells [26]. Furthermore, 1 impaired tumor proliferation of osteosarcoma cells as well, by blocking cell cycle in G1 or G2/M phases, and induced osteoblastic differentiation through specific re-expression of genes that regulate this physiological process [28]. Together with 1 two other SGI-1027 analogues, the bis-quinoline 2 and the bis-pyrimidine 3 (Figure 2) [26] were disclosed as novel DNMTi that, although less potent than 1 in biochemical and cellular assays, were considered structurally worthy of a next step of SAR investigation. Last, the further SGI-1027 analogue 4 (MC3353, Figure 2), in which a benzyloxycarbonyl (*Z*) group replaced the pyrimidine moiety, has been described by us to arrest cell proliferation in lymphoma as well as in solid cancer cells at sub- to single digit micromolar concentrations [29].

**Figure 3:**
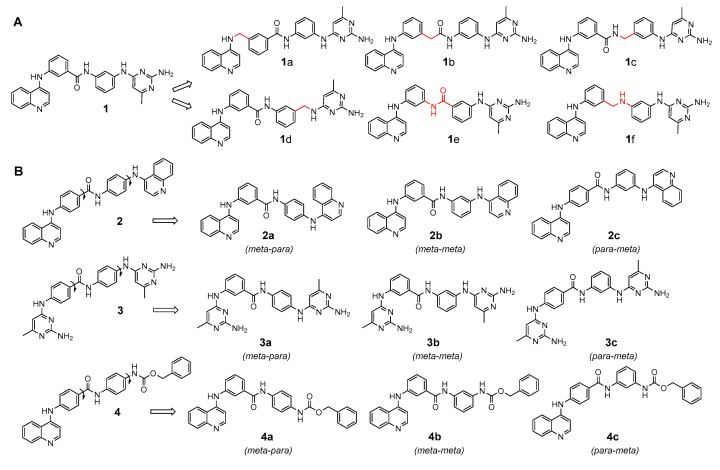
(**A**) Chemical structures of novel analogues of **1** (compounds **1a–f**, in red the changes with respect to **1**). (**B**) Chemical structures of the **2–4** regioisomers (**2a–c**, **3a–c**, and **4a–c**).

Starting from these findings, we applied structural changes to 1 by pin-point methylene spacer insertion at four different points of the molecule (1a–d) to increase its flexibility, and we transformed the amide function of 1 into its reverse amide (1e) or the corresponding amine (1f) linkage (Figure 3A). Afterwards, we performed a regioisomeric study on the bis-quinoline 2, on the bis-pyrimidine 3, and on the *Z* group-containing 4 by synthesizing for each prototype the *meta*-*para*, *meta*-*meta* and *para*-*meta* isomers (compounds 2a–c, 3a–c, and 4a–c, respectively) (Figure 3B). The newly synthesized compounds 1a–f, 2a–c, 3a–c, and 4a–c were screened against human DNMT1 (hDNMT1) and the C-terminal catalytic domain of human DNMT3A (hDNMT3A) to determine their inhibitory activities. Then, all compounds were tested against U937 acute myeloid leukemia (AML) and HL60 acute promyelocytic leukemia (APL) cell lines to detect their antiproliferative effect, and for the most potent compounds (2a–c and 4a–c) the cell death mechanism (apoptosis through the sub-G1 peak, caspase activation, and annexin V induction) in U937 cells was evaluated. Afterwards, 2a–c and 4a–c were tested in a panel of solid cancer cell lines to ascertain their anticancer potential. To confirm that the phenotypic effect observed in cancer cells is related to the activity of such compounds on DNMTs, 2a–c and 4a–c were evaluated in a promoter demethylating and gene re-expression (enhanced green fluorescence protein (EGFP) induction) assay in HCT116 colorectal carcinoma cells. Moreover, since a certain discrepancy between the biochemical and the antiproliferative cellular values of the most potent compounds has been noted, selected compounds 2b and 4c were tested in HCT116 cells to check their ability to degrade the DNMT proteins, highlighting the involvement of a proteasome-dependent molecular mechanism for such protein.

## 2. Results

### 2.1. Design and Synthesis of Quinoline-Based DNMTi

**Scheme 1:**
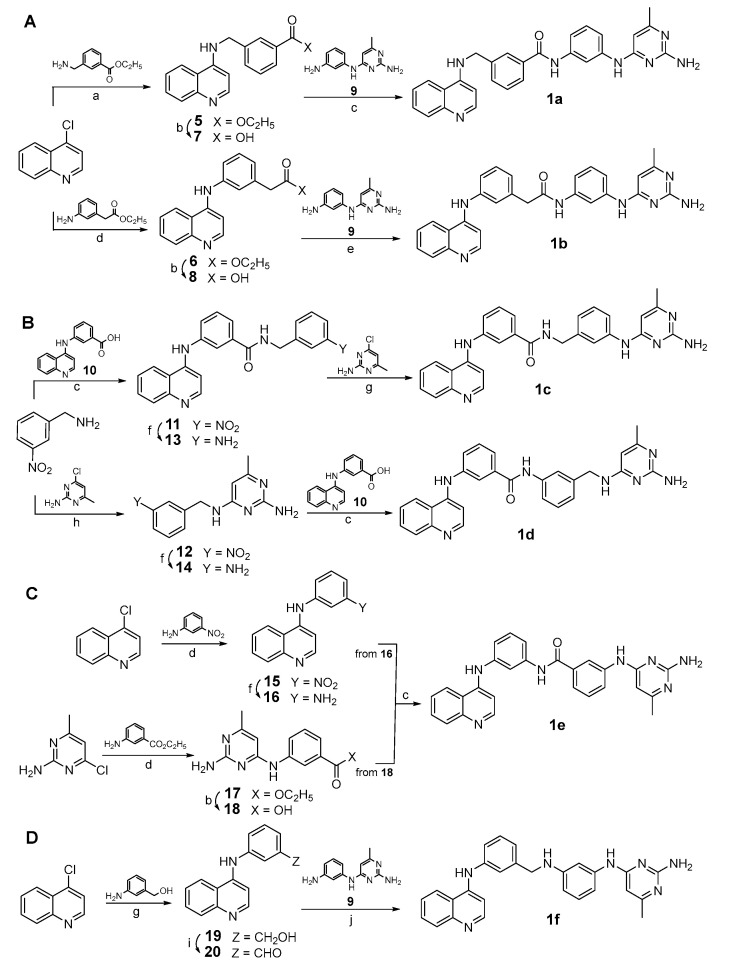
(**A**–**D**) Synthesis of **1a–f**. Reagents and conditions: (**a**) sodium acetate, H_2_O, reflux, 25%; (**b**) 2N potassium hydroxide, ethanol/H_2_O 1:1, rt, 12 h, 78% to 91%; (**c**) triethylamine, benzotriazol-1-yl-oxytripyrrolidino phosphonium hexafluorophosphate (PyBop), dry DMF, N_2_ atmosphere, rt, 3 h, 36%; (**d**) 37% hydrochloric acid, ethanol, 80 °C, 1 h, 70%; (**e**) *N*-ethyl-*N*’-(3-dimethylaminopropyl)carbodiimide hydrochloride (EDCI), triethylamine, dry DCM, room temperature, 48 h, 36%; (**f**) stannous chloride dihydrate, 37% HCl, ethanol, 80 °C, 5 h, 62%; (**g**) 37% HCl, *n*-butanol, 80 °C, 1 h, 46%; (**h**) *N*,*N*-diisopropylethylamine (DIPEA), DMSO, 160 °C, microwave irradiation, 45 min, 80%; (**i**) manganese dioxide, dry THF, 60 °C, 12 h, 48%. (**j**) sodium triacetoxyborohydride, dry DCE, reflux, 10 h, 50%.

**Scheme 2:**
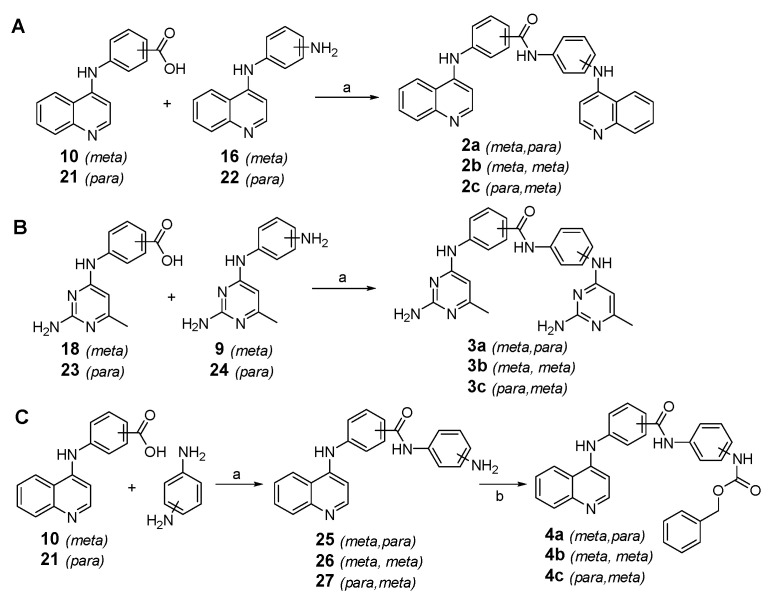
(**A**–**C**) Synthesis of **2a–c**, **3a–c** and **4a–c**. Reagents and conditions: (**a**) triethylamine, PyBop, dry DMF, N_2_ atmosphere, rt, 1–12 h. (**b**) benzyl chloroformate, triethylamine, dry THF, 0 °C, 2 h, 27% to 75%.

Compounds 1a–f, 2a–c, 3a–c, and 4a–c were prepared as summarized in Scheme 1 and Scheme 2. Reaction of 4-chloroquinoline with ethyl 3-(aminomethyl)benzoate in the presence of sodium acetate and water or, alternatively, with ethyl 2-(3-aminophenyl)acetate with aqueous 37% hydrochloric acid (HCl) solution in ethanol furnished the ethyl esters 5 and 6, respectively, which underwent basic hydrolysis to give the corresponding carboxylic acids 7 and 8. Further coupling of 7 and 8 with the *N*^4^-(3-aminophenyl)-6-methylpyrimidine-2,4-diamine 9 [26] led to 1a and 1b, respectively (Scheme 1A). Reaction of 3-nitrobenzylamine with 3-(quinolin-4-ylamino)benzoic acid 10 [26] or, alternatively, with 4-chloro-6-methylpyrimidin-2-amine in the presence of *N*,*N*-diisopropylethylamine at 160 °C in DMSO by microwave irradiation yielded the nitrophenyl derivatives 11 and 12, respectively, which were in turn reduced with stannous chloride dihydrate and 37% HCl in ethanol to the corresponding anilines 13 and 14. Final reaction of 13 with 4-chloro-6-methylpyrimidin-2-amine in the presence of 37% HCl in *n*-butanol or, alternatively, of 14 with 3-(quinolin-4-ylamino)benzoic acid 10 [26] in the presence of triethylamine and benzotriazol-1-yl-oxytripyrrolidinophosphonium hexafluorophosphate (PyBop) in dry DMF under N_2_ atmosphere furnished 1c and 1d, respectively (Scheme 1B). The synthesis of the reverse amide 1e was accomplished by coupling two synthons, *N*^1^-(quinolin-4-yl) benzene-1,3-diamine 16 and 3-((2-amino-6-methylpyrimidin-4-yl) amino) benzoic acid 18, in the presence of triethylamine and PyBop in dry DMF under N_2_ atmosphere. The diamine 16 was prepared by treating 4-chloroquinoline with 3-nitroaniline in the presence of 37% HCl in ethanol and subsequent reduction of the nitro derivative 15 with stannous chloride dihydrate in 37% HCl, while the acid 18 was obtained by reaction between 4-chloro-6-methylpyrimidin-2-amine and ethyl 3-aminobenzoate in 37% HCl followed by alkaline hydrolysis of the resulting ethyl ester 17 (Scheme 1C). Finally, 1f was synthesized by treating 4-chloroquinoline with 3-aminobenzyl alcohol. Further oxidation of the obtained intermediate 19 with manganese dioxide in anhydrous tetrahydrofuran at 60 °C afforded the aldehyde 20 that, after reductive amination with *N*^4^-(3-aminophenyl)-6-methylpyrimidine-2,4-diamine [26] in the presence of sodium triacetoxyborohydride in anhydrous dichloroethane, yielded the final amine 1f (Scheme 1D).

The above reaction performed in the presence of triethylamine and PyBop in dry DMF under nitrogen atmosphere was used to prepare the bis-quinoline (2a–c) and the bis-pyrimidine (3a–c) regioisomers, through reaction of the appropriate synthons (3- and 4-(quinolin-4-ylamino)benzoic acids (10, 21) [26] and *N*^1^-(quinolin-4-yl)benzene-1,3- (16) and -1,4-diamine (22) [26] for 2a–c, Scheme 2A; 3- (18) and 4- (23) -((2-amino-6-methylpyrimidin-4-yl)amino)benzoic acids [26] and *N*^4^-(3- and 4-aminophenyl)-6-methylpyrimidine-2,4-diamines (9, 24) [26] for 3a–c, Scheme 2B). Finally, 10 and 21 were treated with 1,3- and 1,4-phenylendiamine to furnish the amidoanilines 25–27, which were in turn acylated with benzyloxycarbonylchloride to afford the regioisomers 4a–c (Scheme 2C).

### 2.2. Enzyme Assays and Antiproliferative Activities Against U937 AML and HL60 APL Cells

**Figure 4:**
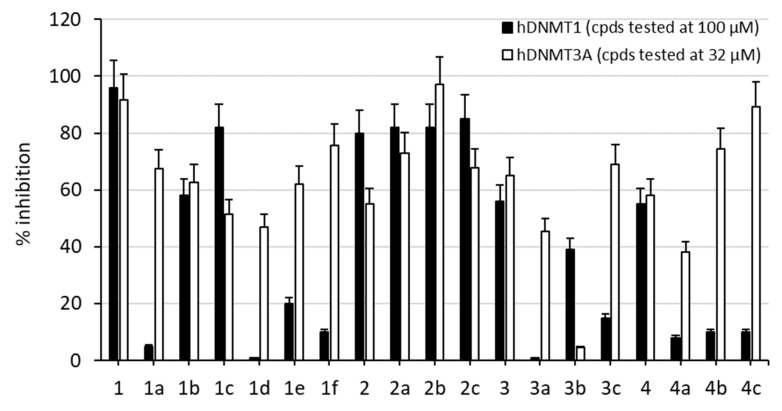
Percentage of inhibiting activities of **1a–f**, **2a–c**, **3a–c**, and **4a–c** against hDNMT1 (compounds tested at 100 µM) and hDNMT3A (compounds tested at 32 µM). Compounds **1–4** were used as reference drugs.

The new compounds 1a–f, 2a–c, 3a–c, and 4a–c were tested at 100 µM against human DNMT1 (hDNMT1), and at 32 µM against the C-terminal catalytic domain of human DNMT3A (hDNMT3A), due to the greater sensitivity of hDNMT3A towards the relative prototypes, and their inhibitory activities have been evaluated. Compounds 1–4 were used as reference controls. As expected, the described new quinoline and pyrimidine derivatives were in general more potent against hDNMT3A than against hDNMT1 (Figure 4).

The EC_50_ values for selected compounds against hDNMT1 and hDNMT3A have been determined and are reported in Table 1. The values of SGI-1027 were added for comparison. 

Table 1 clearly shows that all the more flexible analogues of 1 (1a–d), obtained by introduction of a methylene group in different parts of the molecule, as well as the reverse amide **1e** and the amine 1f displayed much lower hDNMT1 inhibiting potency than 1, and also against hDNMT3A 1a–f showed a drop of 17 to 73-fold of inhibition. Differently, the bis-quinoline regioisomers 2a–c exhibited comparable inhibition towards hDNMT1 as 2, and gained potency against hDNMT3A, with the *meta–meta* isomer 2b being the most potent (EC_50_ = 0.8 µM).

In the case of the bis-pyrimidine analogues, 3a and 3b were less potent or totally inactive against both the hDNMTs with respect to 3, while the *para*-*meta* isomer 3c showed slightly higher hDNMT3A inhibition. Finally, the *Z*-containing 4a–c were less effective than 4 against hDNMT1, and against hDNMT3A the *meta*-*para* isomer 4a displayed a drop of potency, maybe due at least in part to solubility issue, while the *para*-*meta* analog 4c was 3-fold more potent.

In addition to enzyme assays, the antiproliferative activities of 1a–f, 2a–c, 3a–c, and 4a–c against the U937 AML and HL60 APL cell lines have been determined by the MTT method after 48 h treatment (Table 1). IC_50_ values for SGI-1027 and 1–4, used as reference compounds, are reported for comparison. Among the analogues of 1, in U937 cells 1a displayed 5-fold higher potency than 1 in growth arrest induction, while 1e and 1f exhibited just little increase of potency (1.4/1.5-fold). Against HL60 cells, only 1f was more effective than 1. In general, a decrease in DNMT inhibitory potency of such compounds corresponds to lower effects in block of U937 and/or HL60 cell proliferation. Consistently, the regioisomers 2a–c and 4b,c, which were more potent than the prototypes 2 and 4 against DNMT3A, showed antiproliferative effects at submicromolar or single-digit micromolar levels against both the tested leukemia cell lines. In particular, the *meta*-*para* isomer 2a provided the strongest proliferation inhibition in both U937 (IC_50_ values = 0.7 µM) and HL60 (IC_50_ values = 0.2 µM) cells, similar as or better than SGI-1027 and 2. Among the regioisomers of 4, the most potent DNMT inhibitor 4c is also very potent against the leukemia cell lines (IC_50_ values = 1.2 (U937) and 0.3 (HL60) µM). Surprisingly, high potency against both the two tested leukemia cell lines was displayed also by 4a (IC_50_ values = 0.5 (U937) and 0.3 (HL60) µM), which showed a drop of inhibition potency against DNMT3A. This could be due to solubility problems that negatively affected the enzyme assay and/or to the presence of off-target effects. Differently from 2a–c and 4a–c, the bis-pyrimidines 3a–c were practically inactive at cellular level (both cell lines), similarly to their reference compound 3.

Compounds 2b and 4c, chosen as representative samples of 2a–c and 4a–c, were tested against peripheral blood B lymphocyte AHH1 cells to assess their differential toxicity. After 48 h treatment, the two compounds exhibited IC_50_ values of 3.3 (2b) and 9.6 µM (4c), thus showing 2- to 30-fold selectivity for arrest of proliferation in leukemia.

### 2.3. Target Modulation by 2a, 4a, and 4c in KG-1 Leukemia Cells

To check their ability to reactivate gene expression through demethylation in cells, 2a, 4a and 4c were tested in KG-1 acute myeloid leukemia cells at increasing doses (0.5, 1.0, 5.0, 10.0, and 25.0 µM) using a stably integrated luciferase reporter system under the control of a cytomegalovirus (CMV) promoter inhibited by DNA methylation (CMV-luc assay, Table 2), already reported by us [24,29]. SGI-1027 and DAC were used as reference compounds. In this assay, 2a and 4c confirmed their capability to inhibit DNMTs in cells by strong induction of the luciferase signal starting from 5 µM (lower reactivation at higher doses is due to high cytotoxicity of compounds against KG-1 cells), while 4a failed in this test confirming its poor (if any) effect on DNMTs. SGI-1027 displayed the same behavior as 2a and 4c, while DAC was able to reactivate the CMV promoter already at 0.5 µM.

### 2.4. Compounds 2a–c and 4b Induce Apoptosis in U937 Leukemia Cells

**Figure 5:**
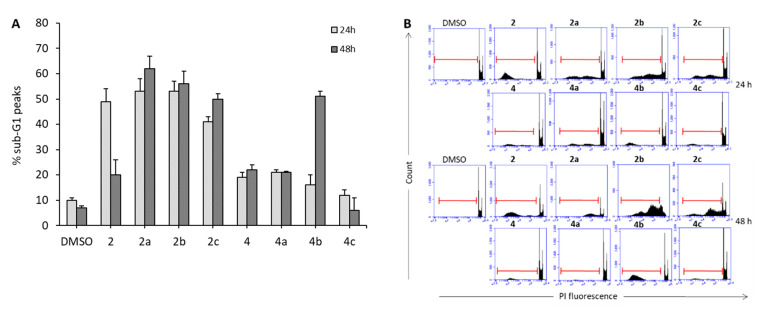
(**A**) Percentage of sub-G1 peaks in U937 cells after treatment with **2a–c**, **4a–c** and their respective references compounds **2** and **4**, all tested at 1 µM for 24 and 48 h. (**B**) FACS panel relative to the sub-G1 peaks.

**Figure 6:**
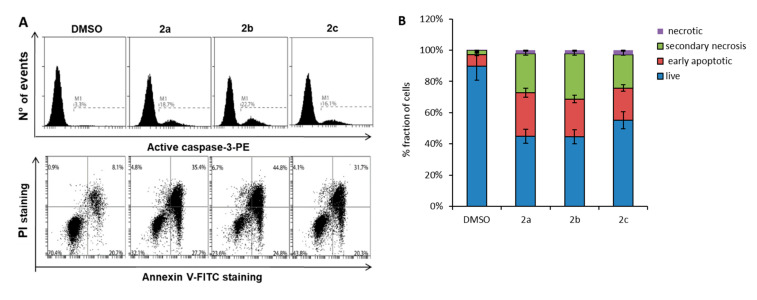
(**A**) Compounds **2a–c** induced caspase 3 activation (up) and annexin V-FITC staining positive cells (down) in U937 cells after treatment at 1 µM for 24 h. (**B**) Percentage of fraction of cells in life, early apoptosis, secondary necrosis, and necrosis.

To characterize the mechanism of the 2a–c and 4a–c antiproliferative activities in leukemia U937 cells, we checked for the ability of such compounds to induce apoptosis. U937 cells were treated with 1 µM 2a–c and 4a–c for 24 and 48 h, and the percentages of sub-G1 peaks in their cell cycle profiles were analyzed as shown in Figure 5. Among the tested compounds, 2a–c as well as 4b induced a remarkable increase of the sub-G1 peaks when compared to untreated cells, whereas 4a and 4c exhibited very low (4a) or no (4c) effect in this assay. Notably, 2a–c were the most potent in sub-G1 induction by providing values up to 62% (2a, 48 h), indicating that apoptosis induction is the main mechanism by which they produce U937 cell proliferation arrest. To confirm this finding, 2a–c were tested in U937 cells at 1 µM for 24 h to check their abilities to induce caspase 3 activation and annexin-V-FITC staining. Consistently with the effects observed with sub-G1 peak induction, 2a–c provided 16% to 23% of active caspase 3 induction, as well as 32% to 45% annexin-V-FITC staining positive cells in U937 cells (Figure 6).

### 2.5. Effects of 2a–c and 4a–c on Solid Cancer Cell Proliferation

We selected 2a–c and 4a–c to evaluate their capability to affect cell growth in a panel of solid cancer cell lines from different origins, i.e., H1299 human non-small cell lung carcinoma, HCT116 human colorectal carcinoma, HeLa epithelial cervix carcinoma, M14 human melanoma, HT1080 human fibrosarcoma, and MCF-7 human breast adenocarcinoma, after 48 h treatment using the MTT method (Table 3). In these assays, the three bis-quinoline isomers 2a–c exhibited higher potency than the prototype 2, displaying IC_50_ values ranging from 0.14 (2a, HeLa cells) to 15 (2a, H1299 cells) µM (IC_50_ values for 2: from 11 (H1299 cells) to 80 (M14 cells) µM). In particular, 2a and 2b showed a general better profile in terms of potency against solid cancer cells, 2a displaying submicromolar IC_50_ values against four cancer cell lines (HCT116, HeLa, M14, HT1080) out of six, and 2b exhibiting the highest potency (IC_50_ = 0.3 µM) against MCF-7 cells, correlating with the strongest inhibition of DNMTs. Differently, among 4a–c, only 4c exerted single-digit micromolar potency against three out of four tested cell lines (HeLa, M14, and HT1080), the others being markedly less effective, in agreement with a weaker DNMT3A inhibition activity.

### 2.6. Target Modulation by 2a–c and 4a–c in HCT116 Cells

**Figure 7:**
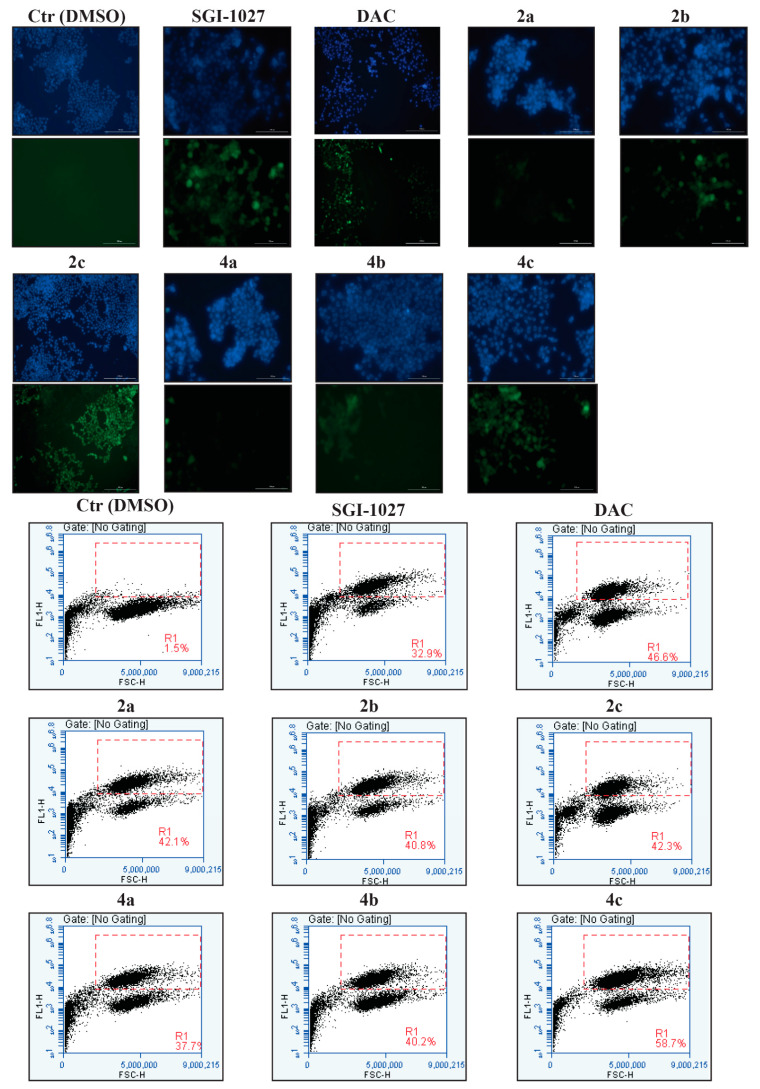
Compounds **2a–c** and **4a–c** display DNA demethylating activity in human HCT116 colon cancer cells. DAPI (up, blue pictures), fluorescence imaging (up, green pictures) and FACS evaluation (down) of HCT116 cells transfected with methylated pUCHL1 vector and treated for five days with DMSO as a vehicle control (Ctr), with DAC (5 µM) and SGI-1027 (0.5 µM) as reference compounds, and with **2a,b** and **4a–c** used at 0.5 µM, and **2c** used at 0.1 µM.

Demethylating activity of 2a–c and 4a–c compounds was also evaluated in HCT116 cells transfected with methylated UCHL1 promoter (pUCHL1, [29,30]). Cells were treated for five days with 2a–c and 4a–c (all at 0.5 µM, except for 2c used at 0.1 µM) and with the positive controls DAC (5 µM) and SGI-1027 (0.5 µM). Fluorescence signal was detected as a measure of demethylating capability. Both fluorescence imaging of live cells and FACS analyses (Figure 7) showed that the treatment with all the tested compounds caused a strong demethylation of pUCHL1, leading to a robust induction of EGFP expression. Specifically, the bis-quinoline isomers 2a–c showed an important demethylation activity around 40%, with 2c displaying the highest potency (42.3%) at the lowest concentration (0.1 µM). Notably, the demethylation capability of 2a–c was more effective than SGI-1027 (32.9%) used at the same concentration, and similar to DAC (46.6%) used at 10-fold higher concentration. Similar effects were observed for 4a–c. Among them, 4c was the strongest demethylating compound with a value of 58.7%, accounting for its high DNMT3A inhibition capability shown in the in vitro enzyme assay.

### 2.7. Selected Compounds 2b and 4c Induce a Downregulation of DNMT Proteins

**Figure 8:**
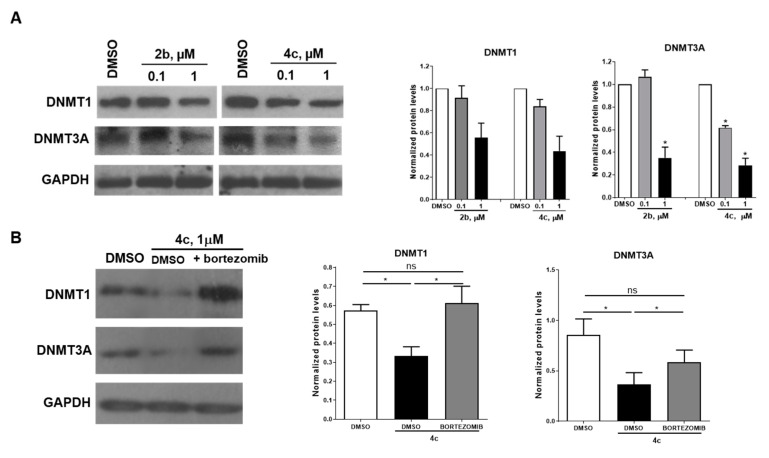
(**A**) Left: Western blot analysis of DNMT1 and DNMT3A protein expression levels in HCT116 cells exposed for 24 h to the indicated concentrations of **2b** or **4c**. Control cells were treated with the same volume of vehicle (DMSO). GAPDH was used as loading control. Blots are representative of two independent experiments. Right: Densitometric analysis of protein levels is reported. (**B**) Left: Western blot analysis of DNMT1 and DNMT3A protein expression levels in HCT116 cells exposed for 24 h to **4c** at 1 µM and co-treated with bortezomib (when indicated) used at 10 nM. Control cells were treated with the same volume of vehicle (DMSO). GAPDH was used as a loading control. Blots are representative of three independent experiments. Right: Densitometric analysis of protein levels is reported. Data are represented as mean ± SEM. Significance is represented as * *p* < 0.05 related to the control.

Prompted by the evidence that 1 decreased DNMT expression in primary osteosarcoma cells [28], and 4 phenocopied similar effects in HCT116 and PC-3 cells [29], we tested selected compounds 2b and 4c as representative samples of the 2 and 4 series to study their effects on DNMT1 and DNMT3A protein expression. In this regard, HCT116 cells were treated with vehicle (DMSO) or with 2b or 4c at different doses (0.1 and 1 µM) for 24 h, and the protein samples were analyzed by western blot. Figure 8A shows that the treatment with the two compounds induced a decrease of both DNMT1 and DNMT3A protein levels at 1 µM, with a higher effect on DNMT3A with respect to DNMT1. This result correlates with the greater inhibition potency of the compounds against the DNMT3A isoform. Compound 4c showed the strongest effect against DNMT3A, since a decrease of the protein expression is detectable also at the lowest dose (0.1 µM). To investigate the mechanism related to this DNMT downregulation, HCT116 cells were treated with 4c (1 µM) alone or in combination with the proteasome inhibitor bortezomib (10 nM). The western blot analysis (Figure 8B) clearly shows that the 4c-dependent decrease of DNMT protein levels occurred via proteasome degradation, since the addition of bortezomib rescued the expression of both the DNMT proteins.

### 2.8. Off-Target Effects: Potential DNA G-Quadruplex (G4) Stabilization and Kinase Inhibition

In addition to the capability to downregulate DNMT proteins, 2a–c and 4a–c were tested to evaluate their eventual DNA G4 stabilizing properties, in analogy to some quinoline compounds reported to bind and stabilize DNA G4 regions involved in repression of transcription at the promoter of oncogenes [31,32,33,34]. Compounds 2a–c and 4a–c were tested at 5 and 10 µM by fluorescence resonance energy transfer (FRET) method with two DNA sequences (F21T and KRAS21R). TMPyP4 [35] (1 µM) was used as the positive control, and SGI-1027, 2 and 4 were added for comparison. However, none of the new compounds displayed a noteworthy increase of the G4-DNA stabilization temperatures (ΔTm values) up to 10 µM (Tables S2 and S3 in Supplementary Materials), thus ruling out the G4 binding as an off-target effect for 2a–c and 4a–c.

Since the quinoline nucleus is a well-known privileged scaffold for human kinases inhibition [36], the bis-quinoline *meta-meta* isomer 2b was tested against a panel of 45 human kinases at fixed 10 µM dose, to explore if inhibition of kinases could represent an additional effect for its antiproliferative and pro-apoptotic properties (Table S4 in Supplementary Materials). The results indicated that 2b inhibited at values ranging from 70% to 98% the biochemical activity of IKK-α (70%), IRK (86%), PKC-β (79%), RAF-1 (79%), Src (94%), TRKA (98%) kinases, all of them found involved in leukemia initiation and development [37,38,39,40,41,42]. This effect, together with DNMT inhibition and induction of protein degradation, could contribute to explain the potency of 2b in proliferation arrest and apoptosis induction.

## 3. Discussion

Starting from the template of SGI-1027 [21], a reliable non-nucleoside DNMTi effective against cancer, we previously identified the regioisomer (*meta*-*meta* isomer) 1 [26,28], the bis-quinoline 2 and the bis-pyrimidine 3 [26] analogues as well as compound 4, in which the *Z*-group replaced the SGI-1027 pyrimidine moiety [29] as new DNMTi active in cancer cells. Here, we report the design, synthesis, and antileukemia (U937 AML and HL60 APL cells) activities of a series of less rigid derivatives of 1, compounds 1a–d, obtained by insertion of a methylene spacer in various points of the molecule of 1, joined to the reverse amide 1e and the amine 1f. Afterwards, we prepared and tested in leukemia some regioisomers of 2–4, specifically 2a–c, 3a–c, and 4a–c, in which a *meta*-*para* (a), *meta*-*meta* (b), or *para*-*meta* (c) linkage has been applied between the two main moieties of the respective prototypes. Among the new compounds 1a–f, 2a–c, 3a–c, and 4a–c, only the bis-quinoline 2a–c and the *Z*-containing 4b,c regioisomers displayed remarkable DNMT (DNMT3A) inhibition and strong antiproliferative effect in U937 and HL60 cells. Compound 4a, although less potent against DNMT3A in enzyme assay, exhibited high leukemia cells’ growth arrest. In U937 cells, this arrest of proliferation was mainly due to induction of apoptosis, as demonstrated by the high sub-G1 peaks shown by U937 cells treated with 2a–c and 4b, and by induction of caspase 3 activation and annexin-V-FITC staining positive cells from 2a–c in the same cell line. When tested against a panel of solid cancer cell lines, 2a–c exhibited cell growth arrest at low to submicromolar levels, whereas 4a–c were less effective. In colon HCT116 cells, 2a–c and 4a–c induced EGFP gene expression through UCHL1 promoter demethylation from 37.7% to 58.7%, demonstrating their demethylating activity in cells. Finally, 2b and 4c, chosen as prototypes of the bis-quinoline and *Z*-containing isomers series, have been proved to induce DNMT, mainly DNMT3A, protein degradation in HCT116 cells with a proteasome-dependent effect, since the combination of 4c with bortezomib, a proteasome inhibitor, rescued in part the decrease of the DNMT protein levels. 

## 4. Materials and Methods

### 4.1. Chemistry

Melting points were determined on a Buchi 530 melting point apparatus and are uncorrected. ^1^H NMR spectra were recorded at 400 MHz on a Bruker AC 400 spectrometer; chemical shifts are reported in δ (ppm) units relative to the internal reference tetramethylsilane (Me_4_Si). All compounds were routinely checked by TLC and ^1^H NMR. TLC was performed on aluminum-backed silica gel plates (Merck DC, Alufolien Kieselgel 60 F254) with spots visualized by UV light. All solvents were reagent grade and, when necessary, were purified and dried by standard methods. Concentration of solutions after reactions and extractions involved the use of a rotary evaporator operating at reduced pressure of ca. 20 Torr. Organic solutions were dried over anhydrous sodium sulfate. Elemental analysis has been used to determine the purity of the final compounds 1a–f, 2a–c, 3a–c, and 4a–c, that is >95%. Analytical results are within ±0.40% of the theoretical values (Table S1, Supplementary Materials). All chemicals were purchased from Sigma-Aldrich, Milan (Italy), or from Alfa Aesar, Karlsruhe (Germany), and were of the highest purity. All the chemical procedures are reported as Supplementary Materials.

### 4.2. DNA Methyltransferase Assays

#### 4.2.1. DNMT1 Assay

His-DNMT1 (182 kDa, human) was cloned, expressed, and purified as described by Lee et al. [43]. The DNMT1 assay was performed according to Gros et al. [44]. Briefly, the reaction is performed with DNMT1 (90 nM in a total volume of 10 µL) treated with the inhibitors used at the desired concentration, 1.25 µM of SAM/[methyl-^3^H] SAM (3TBq/mmol) mix in a ratio of 3:1, and 0.3 µM of biotinylated DNA duplex. The reaction was incubated 2 h at 37 °C, then 8 µL of the solution are transferred into a streptavidin 96-well scintillant coated Flashplate containing 190 µL of 20 mM SAH in 50 mM Tris–HCl (pH 7.4). The Flashplate is agitated for 1 h at room temperature and washed. The plate is read with TopCount (PerkinElmer). In this assay, the negative and positive controls were defined as wells without enzyme and wells with only DMSO, respectively. 

#### 4.2.2. DNMT3A Assay

DNMT3A enzyme inhibition was adapted from the restriction-based fluorescence assay protocol described in Ceccaldi et al. [45]. Briefly, a biotin-labelled oligonucleotide at the 5’ end is hybridized to his complementary strand labelled at the 3’ end with 6-carboxyfluorescein. The DNA duplex containing only one single CpG site overlapping with a restriction site of a methylation sensitive restriction enzyme is transferred in the wells coated with avidin. The methylation reaction is performed by adding human C-terminal DNMT3A at the final concentration of 4 ng/µL in a total volume of 50 µL in the presence of the inhibitors at various concentrations and the co-substrate SAM at the final concentration of 20 µM. The plate was incubated for 1 h at 37 °C, washed, and added with the methylation sensitive restriction enzyme HpyCH4IV, incubated for 1 h further at 37 °C, washed and the fluorescence measured with a PerkinElmer Envision Multilabel Plate Reader. The data are expressed as percentage of inhibition vs. log concentration (M). Data are normalized referring to the “restriction control” (wells coated with labelled duplex not treated nor exposed to DNMT3A, but only cleaved by HpyCH4IV) as the maximum of inhibition (100%) and to the “DMSO control” (wells coated with labelled duplex but treated just with 0.1% DMSO exposed to DNMT3A and then to HpyCH4IV) as the minimum of inhibition (0%; total methylation). 

### 4.3. CMV-luc Assay in KG-1 Cells

The assay was carried out as described by Rilova et al. [46]. The KG-1 cell line was stably transfected with the luciferase firefly (Luc^+^ from pGL3 by Promega, Madison, WI, USA) reporter gene under the control of a methylated CMV promoter (from pEGFP-N1 by Clontech) and selected for the maintenance of the methylation of CMV and luciferase expression silencing (KG-1 CMV-luc). The stably integrated KG-1 CMV-luc cells are cultivated in RPMI-1640 medium (Lonza, Strasbourg, France), supplemented with 10% FCS (Lonza, Strasbourg, France) and 0.5 mg/mL of geneticin (Sigma-Aldrich, Saint-Quentin Fallavier, France), under 5% CO_2_, at 37 °C and seeded at 20,000 cells per well in 96-well plates. After 5 or 24 h of incubation in the presence of compounds or the solvent DMSO, the induction of the promoter is measured by quantification of luciferase with the Brite-lite assay system (PerkinElmer, Villebon-sur-Yvette, France) according to the manufacturer’s protocol. The luminescence is measured on an EnVision Multilabel Plate Reader (PerkinElmer, Villebon-sur-Yvette, France), and the data are expressed as fold induction compared to DMSO control. The mean of 2–4 experiments and its standard error are reported.

### 4.4. UCHL1 Promoter Demethylation Assay (Luciferase Activity Induction) in HCT116 Colon Cancer Cells

The assay was performed as described in [29]. Briefly, human colon cancer HCT116 (ATCC, VA, USA) cells were grown in Dulbecco’s Modified Eagle Medium (DMEM; Euroclone, Milan, Italy) supplemented with 10% fetal bovine serum (FBS; Euroclone, Milan, Italy), 2 mM l-glutamine (Euroclone), and antibiotics (100 U/mL penicillin, 100 g/mL streptomycin) (Euroclone). The pUMLIEP vector (composed by MetLuc-IRES-EGFP construct upon the control of the ubiquitin carboxyl-terminal hydrolase L1 (UCHL1)) was linearized and methylated by with *Cla*I and *Sss*I-methylase enzymes (New England Biolabs, Milan, Italy). After, methylated pUMLIEP was transfected into HCT116 cells following the protocol described in [30]. HCT116 were seeded in 6-well plates in triplicate (2 × 10^4^ per well) and treated every 2 days with 2a-c and 4a–c as well as the relative controls SGI-1027 and DAC, changing the medium. On day 5, cells were observed under by a fluorescent microscope (Cytation 5 Cell Imaging Multi-Mode Reader (BioTeK Milan, Italy) and BD Accuri flow cytometers. Nuclei were counterstained with DAPI (10 µg/mL, Invitrogen, Tournai, Belgium).

### 4.5. Antiproliferative Assays

#### 4.5.1. Cell Lines and Culture Conditions

U937 histiocytic lymphoma, HL60 acute promyelocytic leukemia, H1299 lung adenocarcinoma, MCF7 breast carcinoma, HT1080 fibrosarcoma, M14 melanoma, HeLa epithelial cervix carcinoma and peripheral blood B lymphocyte AHH1 human cell lines were obtained from the Deutsche Sammlung für Mikroorganismen and Zellkulturen (DSZM, Braunschweig, Germany) and were maintained in RPMI 1640 medium contained 10% fetal bovine serum (FBS), 2 mM l-glutamine, and antibiotics in a humidified atmosphere with 5% CO_2_ at 37 °C. HCT116 (ATCC, VA, USA) cells were propagated in Dulbecco’s modified Eagle medium (DMEM) (Euroclone, Milan, Italy) with 10% fetal bovine serum (FBS; Euroclone, Milan, Italy), 2 mM l-glutamine (Euroclone, Milan, Italy), and antibiotics (100 U/mL penicillin, 100 g/mL streptomycin) (Euroclone, Milan, Italy). The experiments were performed with early (3–20) cell passages. Cell lines were treated with compounds at the indicated concentrations in the exponential growth phase.

#### 4.5.2. Cell Proliferation Experiments

To calculate the dose of a compound that causes 50% of cell growth inhibition (IC_50_), exponentially growing cells were seeded in 96-well plates (2 × 10^3^ cells/well) and incubated for 72 h in complete medium. Then, increasing concentrations of different compounds (ranging from 0.01 to 100 μM) were added, and cell proliferation was evaluated by a MTT assay after 48 h. Cells exposed to DMSO were used as control. The effect of compounds was evaluated using the CellTiter-Glo luminescent cell viability assay (Promega, Madison, WI, USA) according to the manufacturer’s instructions. For HCT116, 5 × 10^4^ cells/well were plated in 24-well plate and treated, in duplicate, with compounds at several concentrations for 24 and 48 h of induction. After treatments, MTT solution was added for 3 h at 0.5 mg/mL, the obtained crystals were dissolved in DMSO (Sigma-Aldrich, Milan, Italy), and the absorbance was read at a wavelength of 570 nm with reader TECAN M-200. All the experiments were performed in triplicate and repeated at least three times.

#### 4.5.3. IC_50_ Values Determination and Statistical Analyses

IC_50_ values of compounds against cellular viability were determined by using nonlinear regression fitting curves with GraphPad Prism 8. The t-test used for statistical analyses was performed with GraphPad Prism 8. All the tests were one-tailed and a *p*-value < 0.05 was considered statistically significant.

### 4.6. Flow Cytometric Analysis of Cell Cycle and Apoptosis

Cell cycle analysis was performed on 5 × 10^5^ fixed cells resuspended in a RNase A (3 mg/mL) and propidium iodide (PI) solution (1 mg/mL). Samples were analyzed with a C6 Accuri BD (Becton Dickinson & Co., San Jose, CA, USA). The percentage of apoptosis was measured analyzing sub-G1 peaks. Apoptotic cell death was also evaluated by using annexin V–fluorescein isothiocyanate (FITC) apoptosis kit (BD Bioscience) and assayed according to the manufacturer’s instructions. Cells double-stained for both annexin V and PI were analyzed by flow cytometry. PI was used in conjunction with annexin V–FITC to distinguish cells in the earlier stages of apoptosis (annexin V-FITC positive, PI negative) from those in later stages of apoptosis or that were already dead (annexin V–FITC positive, PI positive). Percentage of cells positive for caspase-3 activation was evaluated by Active Caspase-3 Apoptosis Kit (BD biosciences, Milan, Italy) according to the manufacturer’s instructions.

### 4.7. Western Blot Analyses

Cells were lysed in Laemmli buffer; subsequently the proteins were resolved by sodium dodecyl sulphate-polyacrylamide gel electrophoresis and transferred to a 0.45 µm nitrocellulose membrane (Bio-Rad Laboratories, Hercules, CA, USA). The following primary antibodies were used for immunoblotting: α-DNMT1 (Novus Biologicals, Littleton, CO, USA). α-DNMT3a (SantaCruz Bio-technologies, CA, USA) and α-GAPDH (Millipore Corp., Bedford, MA USA), used as a loading control. The immune complexes were detected with horseradish peroxidase-conjugated species-specific secondary antiserum (Bio-Rad Laboratories, Hercules, CA, USA) then by enhanced chemiluminescence reaction (Bio-Rad Laboratories, Hercules, CA, USA). Densitometric analysis of protein expression was performed by using the Fiji-Image J image processing package. 

## 5. Conclusions

The here described bis-quinolines 2a-c and the *Z*-containing compounds 4a–c represent new SGI-1027 analogues with two simultaneous different activities: (i) high DNMT3A catalytic inhibition, and (ii) capability to degrade the DNMT proteins. Such compounds turned out to be highly active against leukemia (2a–c and 4a–c) and/or solid cancer (2a–c) cell proliferation and are worthy of further investigation in different cancer contexts.

## Figures and Tables

**Table 1:** hDNMT1 and -3A inhibition and antiproliferative activity in U937 AML and HL60 APL cells for **1a–f**, **2a–c**, **3a–c**, and **4a–c**. SGI-1027 and **1–4** were added as reference compounds.

Compd	Enzyme Inhibition (EC_50_ ± SD, µM) ^1^		Antiproliferative Activity (IC_50_ ± SD, µM) ^2^	
	hDNMT1	hDNMT3A	U937	HL60
SGI-1027 ^3^	10 ± 1	0.8 ± 0.1	0.70 ± 0.08	0.70 ± 0.16
**1** ^3^	5.7 ± 0.2	0.7 ± 0.5	6.90 ± 0.8	5.90 ± 0.7
**1a**	ND ^4^	21.0 ± 3.1	1.40 ± 0.2	18.1 ± 2.4
**1b**	ND	19.0 ± 2.0	37.0 ± 5.9	40.0 ± 5.0
**1c**	35 ± 7	31.0 ± 6.0	25.8 ± 3.2	36.0 ± 4.0
**1d**	ND	51.0 ± 10.0	67.0 ± 8.8	67.0 ± 7.4
**1e**	ND	21.0 ± 4.0	4.8 ± 0.6	9.9 ± 1.1
**1f**	ND	12.0 ± 3.0	4.4 ± 0.7	1.9 ± 0.1
**2** ^5^	33 ± 1	27.0 ± 5.0	6.6 ± 0.9	65.0 ± 8.1
**2a**	23 ± 4	6.0 ± 1.0	0.70 ± 0.09	0.20 ± 0.03
**2b**	27 ± 5	0.8 ± 0.1	1.5 ± 0.1	1.6 ± 0.2
**2c**	22 ± 3	5.6 ± 1.0	1.5 ± 0.1	1.9 ± 0.3
**3** ^5^	68 ± 4	18.0 ± 2.0	60.0 ± 8.2	119.0 ± 12.9
**3a**	ND	41.0 ± 10.0	52.4 ± 7.6	83.0 ± 10.2
**3b**	ND	>100	123.0 ± 15.0	225.0 ± 28.5
**3c**	ND	13.0 ± 0.9	66.0 ± 9.2	125.0 ± 16.2
**4** ^6^	67 ± 4	17.0 ± 3.0	2.3 ± 0.4	8.3 ± 1.0
**4a** ^7^	ND	>32	0.50 ± 0.08	0.30 ± 0.06
**4b**	ND	13.0 ± 1.4	3.2 ± 0.6	1.9 ± 0.3
**4c**	ND	6.1 ± 1.0	1.2 ± 0.2	0.30 ± 0.07

^1^ Data are the mean of at least two independent experiments. ^2^ Data are the mean of three independent experiments. ^3^ Reference [27]. ^4^ ND, not determined. ^5^ Reference [26]. ^6^ Reference [29]. ^7^ Precipitation occurred in the medium.

**Table 2:** Fold induction of the luciferase signal of the CMV-luc construct in KG-1 leukemia cells treated with **2a**, **4a**, and **4c**. SGI-1027 and DAC were added as reference compounds.

Compd	Luciferase Expression (Fold ± SD ^1^)				
	0.5 µM	1 µM	5 µM	10 µM	25 µM
**2a**	1.02 ± 0.04	1.05 ± 0.12	19.94 ± 4.26	4.66 ± 1.63	1.59 ± 0.59
**4a**	1.05 ± 0.12	1.02 ± 0.07	1.04 ± 0.16	1.45 ± 0.39	1.60 ± 1.00
**4c**	0.96 ± 0.01	0.87 ± 0.05	40.40 ± 12.75	28.51 ± 3.39	8.07 ± 1.96
SGI-1027	1.07 ± 0.16	1.16 ± 0.20	25.54 ± 5.77	6.00 ± 2.57	3.24 ± 1.66
DAC	18.13 ± 5.52	19.80 ± 3.88	23.82 ± 5.44	20.96 ± 4.02	ND ^2^

^1^ All experiments were performed from *n* = 2 to *n* = 4. ^2^ ND, not determined.

**Table 3:** Antiproliferative IC_50_ values of compounds 2a–c, 4a–c and their respective reference compounds 2 and 4 against a panel of solid cancer cell lines.

Compd	Antiproliferative Activity (IC_50_ ± SD, µM) ^1^					
	H1299	HCT116	HeLa	M14	HT1080	MCF-7
**2**	11.4 ± 1.8	13.3 ± 2.1	52.0 ± 5.7	80.0 ± 9.1	17.0 ± 2.1	ND ^2^
**2a**	14.7 ± 2.3	1.0 ± 0.2	0.14 ± 0.04	0.50 ± 0.08	0.50 ± 0.09	4.1 ± 0.7
**2b**	5.3 ± 0.9	1.5 ± 0.3	2.0 ± 0.3	4.7 ± 0.6	2.7 ± 0.4	0.30 ± 0.06
**2c**	14.0 ± 1.8	2.3 ± 0.5	4.2 ± 0.8	3.8 ± 0.7	2.6 ± 0.6	2.2 ± 0.4
**4**	5.9 ± 0.8	8.6 ± 1.0	3.2 ± 0.5	2.9 ± 0.4	1.7 ± 0.2	ND
**4a**	45.0 ± 4.1	23.9 ± 2.6	77.0 ± 7.9	69.0 ± 8.1	19.5 ± 2.8	ND
**4b**	40.0 ± 4.8	20.5 ± 2.8	65.0 ± 7.3	15.3 ± 2.0	13.1 ± 1.5	ND
**4c**	6.9 ± 0.9	17.7 ± 2.4	7.4 ± 1.2	4.5 ± 0.8	3.9 ± 0.8	ND

^1^ Data are the mean of at least three independent experiments. ^2^ ND, not detected.

## Supplementary Materials

The following are available online at https://www.mdpi.com/2072-6694/12/2/447/s1. Synthetic procedures and chemical and physical data of new compounds from 1a to 27. Table S1: Elemental analysis of the final compounds 1a–f, 2a–c, 3a–c, and 4a–c, Experimental procedure for Fluorescence Resonance Energy Transfer (FRET) melting assay. Table S2: Synthetic oligonucleotides used in FRET experiments. Table S3: ∆Tm values for G-quadruplex DNA FRET assays (F21T and KRAS). Experimental procedure of kinase inhibitory assays. Table S4: Screening of compound **2a** on a panel of kinases.
