# Correction: Zwergel et al. Novel Quinoline Compounds Active in Cancer Cells through Coupled DNA Methyltransferase Inhibition and Degradation. *Cancers* 2020, *12*, 447

**DOI:** 10.3390/cancers16061230

**Published:** 2024-03-21

**Authors:** Clemens Zwergel, Rossella Fioravanti, Giulia Stazi, Federica Sarno, Cecilia Battistelli, Annalisa Romanelli, Angela Nebbioso, Eduarda Mendes, Alexandra Paulo, Raffaele Strippoli, Marco Tripodi, Dany Pechalrieu, Paola B. Arimondo, Teresa De Luca, Donatella Del Bufalo, Daniela Trisciuoglio, Lucia Altucci, Sergio Valente, Antonello Mai

**Affiliations:** 1 Department of Drug Chemistry and Technologies, Sapienza University of Rome, P. le A. Moro 5, 00185 Rome, Italy; clemens.zwergel@uniroma1.it (C.Z.); rossella.fioravanti@uniroma1.it (R.F.); giulia.stazi@uniroma1.it (G.S.); annalisa.romanelli@uniroma1.it (A.R.); 2 Department of Precision Medicine, University of Studi della Campania Luigi Vanvitelli, Vico L. De Crecchio 7, 80138 Naples, Italy; federica.sarno@unicampania.it (F.S.); angela.nebbioso@unicampania.it (A.N.); lucia.altucci@unicampania.it (L.A.); 3 Department of Molecular Medicine, Sapienza University of Rome, Viale Regina Elena 324, 00161 Rome, Italy; cecilia.battistelli@uniroma1.it (C.B.); raffaele.strippoli@uniroma1.it (R.S.); marco.tripodi@uniroma1.it (M.T.); 4 Research Institute for Medicines, Medicinal Chemistry Group, Faculty of Pharmacy, Universidade de Lisboa, 1649 003 Lisbon, Portugal; ermendes@ff.ulisboa.pt (E.M.); mapaulo@ff.ulisboa.pt (A.P.); 5 National Institute for Infectious Diseases L. Spallanzani, IRCCS, Via Portuense, 292, 00149 Rome, Italy; 6 Istituto Pasteur- Fondazione Cenci Bolognetti, Department of Molecular Medicine, Sapienza Università di Roma, 00185 Rome, Italy; 7 ETaC CNRS FRE3600, LMBE, 118 route de Narbonne, 31062 Toulouse, France; dany.pechalrieu@gmail.com (D.P.); paola.arimondo@cnrs.fr (P.B.A.); 8 Epigenetic Chemical Biology, Institute Pasteur, CNRS UMR3523, 28 rue du Docteur Roux, 75724 Paris, France; 9 Preclinical Models and New Therapeutic Agents Unit, IRCCS-Regina Elena National Cancer Institute, Via Elio Chianesi 53, 00144 Rome, Italy; teresa.deluca@ifo.gov.it (T.D.L.); donatella.delbufalo@ifo.gov.it (D.D.B.); 10 Institute of Molecular Biology and Pathology, National Research Council (CNR), Via Degli Apuli 4, 00185 Rome, Italy

In the original publication [1], there was a mistake in Figure 7 as published. Figure 7 contained a duplication of the DAC GFP image (up), taken from the corresponding DAC GFP image published in ref. [1]. As two manuscripts were written with a short time between them, this is the result of an error while copying/pasting the individual pictures used to prepare Figure 7 in this paper. The erroneous picture did not affect the corresponding quantification and the interpretation of the results as this was performed with the correct picture set. The corrected Figure 7 appears below.

The authors apologize for any inconvenience caused and state that the scientific conclusions are unaffected. This correction was approved by the Academic Editor. The original publication has also been updated.

## Figures and Tables

**Figure 7:**
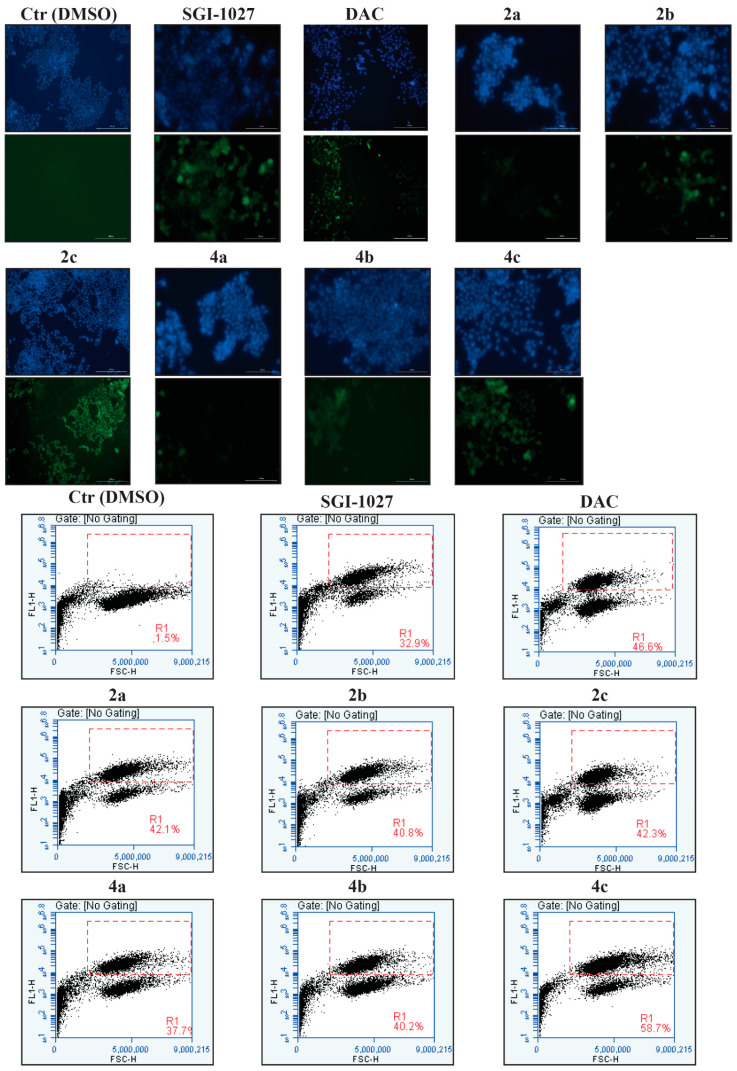
Compounds **2a**–**c** and **4a**–**c** display DNA demethylating activity in human HCT116 colon cancer cells. DAPI (up, blue pictures), fluorescence imaging (up, green pictures) and FACS evaluation (down) of HCT116 cells transfected with methylated pUCHL1 vector and treated for five days with DMSO as a vehicle control (Ctr), with DAC (5 µM) and SGI-1027 (0.5 µM) as reference compounds, and with **2a**,**b** and **4a–c** used at 0.5 µM, and **2c** used at 0.1 µM.

## References

[1] Zwergel C., Fioravanti R., Stazi G., Sarno F., Battistelli C., Romanelli A., Nebbioso A., Mendes E., Paulo A., Strippoli R. (2020). Novel Quinoline Compounds Active in Cancer Cells through Coupled DNA Methyltransferase Inhibition and Degradation. Cancers.

