# Supplementary material for: Novel Quinoline Compounds Active in Cancer Cells through Coupled DNA Methyltransferase Inhibition and Degradation
Source: Cancers (Basel). 2020 Feb 14;12(2):447. doi: 10.3390/cancers12020447 (PMC7073229; doi:10.3390/cancers12020447)
Supplement: Supplementary file 1 [file cancers-12-00447-s001.pdf]

Clemens Zwergel, Rossella Fioravanti, Giulia Stazi, Federica Sarno, Cecilia Battistelli, Annalisa Romanelli, Angela Nebbioso, Eduarda Mendes, Alexandra Paulo, Raffaele Strippoli, Marco Tripodi, Dany Pechalrieu, Paola B. Arimondo, Teresa De Luca, Donatella Del Bufalo, Daniela Trisciuoglio,* Lucia Altucci, Sergio Valente,* and Antonello Mai*

Novel Quinoline Compounds Highly Potent in Cancer Cells Through Coupled DNA Methyltransferase Inhibition and Degradation

Supplementary materials

Contents

Chemistry. Synthetic procedures and chemical and physical data of new compounds **1a** to **27** p. S2

Table S1. Elemental analysis of the final compounds **1a-f**, **2a-c**, **3a-c**, and **4a-c** p. S14

Experimental procedure for Fluorescence Resonance Energy Transfer (FRET) melting assay p. S15

Table S2. Synthetic oligonucleotides used in FRET experiments p. S15

Table S3. Δ*T*m values for G-quadruplex DNA FRET assays (F21T and KRAS) p. S16

Experimental procedure of kinase inhibitory assays p. S17

Table S4. Screening of compound **2a** on a panel of kinases p. S18

Whole original Western Blots p. S21

References p. S23

Chemistry

General Procedure for the Synthesis of Compounds 1a, 1d, 1e, 2a-c, 3a-c, and of the Intermediate Compounds 11 and 25-27.

Triethylamine (2.06 mmol) and benzotriazol-1-yl-oxytripyrrolidinophosphonium hexafluorophosphate (PyBOP) (0.49 mmol) were added to a solution of the appropriate acid (**7** for **1a**, **10**[1] for **1d**, **18** for **1e**, **10**[1] or **21**[1] for **2a-c**, **18** or **23**[1] for **3a-c**, **10**[1] for **11**, and **10**[1] or **21**[1] for **25-27**) (0.41 mmol) in dry DMF (4 mL) under nitrogen atmosphere. The resulting mixture was stirred for 45 min at room temperature; upon activation of the acid, checked by TLC, the corresponding amine (**9**[1] for **1a**, **14** for **1d**, **16** for **1e**, **16** or **22**[1] for **2a-c**, **9**[1] or **24**[1] for **3a-c**, 3-nitrobenzylamine for **11**, 3- or 4-phenylenediamine for **25-27**) (0.41 mmol) was added. After 1 h, the reaction was quenched with distilled water (50 mL), and the precipitate was filtered and washed with distilled water providing the desired pure product.

N-*(3-((2-Amino-6-methylpyrimidin-4-yl)amino)phenyl)-3-((quinolin-4-ylamino)methyl)benzamide (**1a**).* Mp: 153-155 °C; recrystallization solvent: acetonitrile; yield: 36%. ^{1}H NMR (DMSO, 400 MHz) δ 2.09 (s, 3H, CH_3), 4.67 (d, 2H, *J* = 5.6 Hz, CH_2), 5.92 (s, 1H, pyrimidine proton), 6.07 (bs, 2H, NH_2), 6.40 (d, 1H, *J* = 5.2 Hz, quinoline proton), 7.21 (t, 1H, *J* = 8.0 Hz, benzene proton), 7.32 (d, 1H, *J* = 8.0 Hz, benzene proton), 7.49-7.52 (m, 2H, benzene and quinoline protons), 7.59 (t, 2H, *J* = 8.4 Hz, benzene and quinoline protons), 7.67 (t, 1H, *J* = 7.2 Hz, quinoline proton), 7.79-7.86 (m, 2H, benzene protons), 7.99 (s, 2H, benzene protons), 8.14 (bs, 1H, N*H*CH_2), 8.33-8.35 (d, 2H, quinoline protons), 9.03 (bs, 1H, N*H*), 10.15 (bs, 1H, CON*H*) ppm.

*N-(3-(((2-Amino-6-methylpyrimidin-4-yl)amino)methyl)phenyl)-3-(quinolin-4-ylamino)benzamide (**1d**).* Mp: 260-262 °C; recrystallization solvent: methanol; yield: 18%. ^{1}H NMR (DMSO, 400 MHz) δ 2.01 (s, 3H, CH_3), 4.47 (d, 2H, *J* = 5.6 Hz, CH_2), 5.62-5.65 (m, 1H, pyrimidine proton), 5.82 (s, 2H, NH_2),

7.04-7.09 (m, 2H, benzene proton), 7.19-7.21 (m, 1H, benzene proton), 7.27-7.31 (m, 1H, benzene proton), 7.54-7.67 (m, 3H, benzene and quinoline protons), 7.70-7.76 (m, 4H, benzene and quinoline protons) 7.89-7.94 (m, 2H, benzene and quinoline protons), 8.40-8.42 (m, 1H, quinoline proton), 8.51-8.52 (bs, 1H, N*H*), 9.15 (bs, 1H, N*H*), 10.29 (bs, 1H, CON*H*) ppm.

*3-((2-amino-6-methylpyrimidin-4-yl)amino)-N-(3-(quinolin-4-ylamino)phenyl)benzamide (**1e**).* Mp: 227-229 °C; recrystallization solvent: acetonitrile/methanol; yield: 46%. ^{1}H NMR (DMSO, 400 MHz) δ 2.16 (s, 3H, CH_3), 6.00 (s, 1H, pyrimidine proton), 6.67 (s, 2H, NH_2), 6.98-7.01 (m, 1H, benzene proton), 7.16-7.19 (m, 1H, benzene proton), 7.46-7.47 (m, 2H, benzene and quinoline protons), 7.58-7.67 (m, 3H, benzene and quinoline protons), 7.85-7.87 (m, 1H, benzene proton), 7.95-8.05 (m, 3H, benzene and quinoline protons), 8.14-8.19 (m, 1H, quinoline proton), 8.52-8.58 (m, 2H, benzene and quinoline protons), 9.70 (bs, 1H, N*H*), 9.85 (bs, 1H, N*H*),10.42 (bs, 1H, CON*H*) ppm.

*3-(Quinolin-4-ylamino)-N-(4-(quinolin-4-ylamino)phenyl)benzamide (**2a**).* Mp: 235-237 °C; recrystallization solvent: acetonitrile/methanol; yield: 17%. ^{1}H NMR (DMSO, 400 MHz) δ 6.84 (d, 1H, J = 5.6 Hz, benzene proton), 7.06 (d, 1H, J = 5.2 Hz, benzene proton), 7.39–7.42 (m, 2H, benzene protons), 7.59-7.63 (m, 4H, quinoline and benzene protons), 7.76-7.77 (m, 3H, quinoline and benzene protons), 7.88-7.98 (m, 5H, quinoline protons), 8.45-8.54 (m, 4H, quinoline protons), 9.52-9.58 (bs, 2H, N*H*), 10.42 (bs, 1H, CON*H*) ppm.

*3-(Quinolin-4-ylamino)-N-(3-(quinolin-4-ylamino)phenyl)benzamide (**2b**).* Mp: 292-295 °C; recrystallization solvent: methanol; yield: 24%. ^{1}H NMR (DMSO, 400 MHz) δ 7.02-7.13 (m, 3H, benzene protons), 7.39 (t, 1H, J = 8.0 Hz, benzene proton), 7.55-7.59 (m, 5H, benzene and quinoline protons), 7.69-7.78 (m, 3H, benzene and quinoline protons), 7.88-7.99 (m, 4H, benzene and quinoline protons), 8.45-8.52 (m, 4H, quinoline protons), 9.02 (bs, 1H, N*H*), 9.15 (bs, 1H, N*H*), 10.35 (bs, 1H, CON*H*) ppm.

*4-(Quinolin-4-ylamino)-N-(3-(quinolin-4-ylamino)phenyl)benzamide (**2c**).* Mp: >300 °C; recrystallization solvent: methanol; yield: 22%. ^{1}H NMR (DMSO, 400 MHz) δ 7.04 (d, 1H, *J* = 4.4 Hz, benzene proton), 7.10 (d, 1H, *J* = 8.0 Hz, benzene proton), 7.22 (d, 1H, *J* = 4.4 Hz, benzene proton), 7.35–7.37 (m, 1H, benzene proton), 7.45-7.59 (m, 5H, benzene and quinoline protons) 7.61-7.69 (m, 2H, benzene and quinoline protons), 7.88-7.98 (m, 3H, benzene and quinoline protons), 8.02 (d, 2H, J = 8.0 Hz, quinoline protons), 8.38 (d, 1H, *J* = 8.8 Hz, quinoline proton), 8.43 (d, 1H, *J* = 8.0 Hz, quinoline proton), 8.50 (d, 1H, *J* = 4.8 Hz, quinoline proton), 8.59 (d, 1H, *J* = 4.8 Hz, quinoline proton), 9.04 (bs, 1H, N*H*), 9.31 (bs, 1H, N*H*), 10.23 (s, 1H, CON*H*) ppm.

*3-((2-Amino-6-methylpyrimidin-4-yl)amino)-N-(4-((2-amino-6-methylpyrimidin-4-yl)amino)phenyl)benzamide (**3a**).* Mp: 218-220 °C; recrystallization solvent: acetonitrile/methanol; yield: 88%. ^{1}H NMR (DMSO, 400 MHz) δ 2.14 (s, 6H, 2 × CH_3), 6.05 (d, 2H, pyrimidine protons), 6.49 (bs, 2H, NH_2), 6.77 (bs, 2H, NH_2), 7.41 (t, 1H, *J* = 8.4 Hz, benzene proton), 7.57 (d. 1H, *J* = 6.8 Hz, benzene proton), 7.73 (s, 4H, benzene protons), 8.01 (s, 1H, benzene proton), 8.16 (d, 1H, *J* = 7.6 Hz, benzene proton), 9.71 (bs, 1H, N*H*), 9.81 (bs, 1H, N*H*), 10.26 (s, 1H, CON*H*) ppm.

*3-((2-Amino-6-methylpyrimidin-4-yl)amino)-N-(3-((2-amino-6-methylpyrimidin-4-yl)amino)phenyl)benzamide (**3b**).* Mp: 105-107 °C; recrystallization solvent: toluene; yield: 64%. ^{1}H NMR (DMSO, 400 MHz) δ 2.14 (s, 6H, 2 × CH_3), 5.97 (s, 1H, pyrimidine proton), 5.99 (s, 1H, pyrimidine proton), 6.43 (bs, 2H, NH_2), 6.49 (bs, 2H, NH_2), 7.25 (t, 1H, *J* = 7.6 Hz, benzene proton), 7.38-7.45 (m, 2H, benzene protons), 7.54 (d, 1H, *J* = 8.0 Hz, benzene proton), 7.60 (d, 1H, *J* = 7.6 Hz, benzene proton), 7.94 (s, 1H, benzene proton), 8.03 (s, 1H, benzene proton), 8.17-8.19 (d, 1H, *J* = 7.2 Hz, benzene proton), 9.42 (bs, 1H, N*H*), 9.46 (bs, 1H, N*H*), 10.18 (s, 1H, CON*H*) ppm.

*4-((2-Amino-6-methylpyrimidin-4-yl)amino)-N-(3-((2-amino-6-methylpyrimidin-4-yl)amino)phenyl)benzamide (**3c**).* Mp: 292-294 °C; recrystallization solvent: methanol; yield: 41%. ^{1}H NMR (DMSO, 400 MHz) δ 2.09 (s, 3H, CH_3), 2.12 (s, 3H, CH_3), 5.94 (s, 1H, pyrimidine proton), 5.96

(s, 1H, pyrimidine proton), 6.97 (bs, 2H, N*H*$_2$), 6.26 (bs, 2H, N*H*$_2$), 6.97-7.02 (m, 1H, benzene proton), 7.19-7.23 (s, 1H, benzene proton), 7.35-7.41 (m, 1H, benzene proton), 7.87-7.92 (s, 4H, benzene protons), 8.01 (s, 1H, benzene proton), 9.02 (bs, 1H, N*H*), 9.37 (bs, 1H, N*H*), 9.96 (bs, 1H, CON*H*) ppm.

N-*(3-Nitrobenzyl)-3-(quinolin-4-ylamino)benzamide (**11**)*. Mp: 195-198 °C; recrystallization solvent: acetonitrile/methanol; yield: 45%. ^{1}H NMR (DMSO, 400 MHz) 4.56 (d, 2H, *J* = 4.4 Hz, CH_2), 7.43-7.55 (m, 3H, benzene protons), 7.94-8.01 (m, 5H, benzene and quinoline protons), 8.16-8.23 (m, 2H, benzene and quinoline protons), 8.59-8.61 (m, 2H, benzene and quinoline protons), 8.69 (d, 1H, *J* = 8.8 Hz, quinoline proton), 8.79 (d, 1H, *J* = 4.0 Hz, quinoline proton), 8.96 (s, 1H, CONH), 9.08 (bs, 1H, N*H*) ppm.

N-*(4-Aminophenyl)-3-(quinolin-4-ylamino)benzamide (**25**)*. Mp: 150-152 °C; recrystallization solvent: acetonitrile; yield: 98%; ^{1}H NMR (DMSO-d_6, 400 MHz, δ; ppm) δ 5.05 (bs, 2H, NH_2), 6.55 (d, 2H, J= 8.0 Hz, benzene protons), 7.02 (d, 1H, J= 4.0 Hz, quinoline proton), 7.37 (d, 2H, , J= 8.0 Hz, benzene protons), 7.55 (m, 3H, benzene protons), 7.70 – 7.77 (m, 2H, benzene and quinoline protons), 7.91 (s, 2H, quinoline protons), 8.41 (d, 1H, J= 16.0 Hz, quinoline proton), 8.50 (d, 1H, J= 4.0 Hz, quinoline proton), 9.13 (bs, 1H, NH), 9.92 (bs, 1H, NH) ppm.

N-*(3-Aminophenyl)-3-(quinolin-4-ylamino)benzamide (**26**)*. Mp: 132-134 °C; recrystallization solvent: toluene; yield: 89%. ^{1}H NMR (DMSO, 400 MHz) δ 6.32 (d, 1H, *J* = 8.4 Hz, benzene proton), 6.86 (d, 1H, *J* = 8.4 Hz, benzene proton), 6.95-7.02 (m, 2H, benzene and quinoline protons), 7.10-7.13 (m, 1H, benzene proton), 7.59-7.68 (m, 3H, benzene and quinoline protons), 7.75-7.84 (m, 2H, benzene and quinoline protons), 7.93-7.95 (m, 2H, benzene protons), 8.50-8.54 (m, 2H, quinoline protons), 9.70 (bs, 1H, N*H*), 10.00 (s, 1H, CON*H*) ppm.

N-*(3-Aminophenyl)-4-(quinolin-4-ylamino)benzamide (**27**)*. Mp: 145-147 °C; recrystallization solvent: acetonitrile; yield: 55%. ^{1}H NMR (DMSO, 400 MHz) δ 5.03 (bs, 2H, NH_2), 6.45 (d, 1H, *J* = 8.0 Hz,

benzene proton), 7.29-7.42 (m, 3H, benzene and quinoline protons), 7.38 (d, 1H, *J* = 8.0 Hz, benzene proton), 7.57-7.71 (m, 4H, benzene and quinoline protons), 7.95-7.99 (m, 3H, benzene and quinoline protons), 8.09 (d, 1H, *J* = 16.0 Hz, quinoline proton), 8.62 (d, 1H, *J* = 4.0 Hz, quinoline proton), 9.10 (bs, 1H, NH), 9.89 (bs, 1H, CONH) ppm.

Synthesis of *N*-(3-((2-amino-6-methylpyrimidin-4-yl)amino)phenyl)-2-(3-(quinolin-4-ylamino)phenyl)acetamide (1b). The acid **8** (0.18 mmol, 0.05 g), *N*-ethyl-*N'*-(3,3-dimethylaminopropyl)carbodiimide hydrochloride (0.27 mmol, 0.05 g), triethylamine (0.36 mmol, 0.05 mL) and anhydrous dichloromethane (7 mL) were left under stirring for 1 h at room temperature. Then the amine **9**[1] (0.18 mmol, 0.039 g) in anhydrous tetrahydrofuran (1 mL) was added. After 48 h the reaction was quenched by the addition of water (20 mL), saturated aqueous sodium chloride solution (10 mL) and then extracted with ethyl acetate (3 × 20 mL). The combined organic layers were dried over sodium sulphate, filtered and concentrated in vacuo. The crude residue was purified by column chromatography on silica gel eluting with ethyl acetate/methanol 2:1 obtaining the pure compound **1b**. Mp: 163-165 °C; recrystallization solvent: acetonitrile; yield: 40%. ^{1}H NMR (DMSO, 400 MHz) δ 2.03 (s, 3H, CH_3), 3.76 (s, 2H, CH_2), 5.98 (s, 1H, pyrimidine proton), 6.08 (s, 2H, NH_2), 7.01 (s, 1H, quinoline proton), 7.24-7.32 (m, 5H, benzene and quinoline protons), 7.42-7.45 (m, 2H, benzene and quinoline protons), 7.69-7.72 (t, 1H, benzene proton), 7.78-7.80 (t, 1H, benzene proton), 7.98-7.99 (d, 1H, benzene proton), 8.08 (s, 1H, benzene proton), 8.31-8.35 (m, 2H, quinoline protons), 9.13 (bs, 2H, N*H*), 10.22 (s, 1H, CON*H*) ppm.

General Procedure for the Synthesis of 1c and of the Intermediate Compounds 6, 15, 17, and 19. The opportune chloroderivative (4-chloro-6-methylpyrimidin-2-amine for **1c** and **17**, 4-chloroquinoline for **6**, **15**, and **19**) (1.2 mmol), the appropriate amine (**13** for **1c**, ethyl 2-(3-aminophenyl)acetate for **6**, 3-nitroaniline for **15**, ethyl 3-aminobenzoate for **17**, and 3-aminobenzyl alcohol for **19**) (1.2 mmol) and a catalytic amount (2 drops) of aqueous 37% HCl were refluxed in ethanol (for **6**, **15**, and **17**) or in *n*-

butanol (for **1c** and **19**) (7 mL) for 1 h. After cooling, half of the alcohol was distilled out, the obtained solid was filtered and washed twice with a 1:1 mixture of diethyl ether and petroleum ether (5 mL) obtaining the desired product pure as a hydrochloride salt.

N-*(3-((2-amino-6-methylpyrimidin-4-yl)amino)benzyl)-3-(quinolin-4-ylamino)benzamide (**1c**)*. Mp: 183-185 °C; recrystallization solvent: acetonitrile/methanol; yield: 28%. ^{1}H NMR (DMSO, 400 MHz) δ 2.05 (s, 3H, CH_3), 4.47 (d, 2H, *J* = 4.4 Hz, CH_2), 5.87 (s, 1H, pyrimidine proton), 6.07 (s , 2H, NH_2), 6.89 (d, 1H, *J* = 6.4 Hz, quinoline proton), 7.00 (d, 1H, *J* = 4.4 Hz, benzene proton), 7.11 (t, 1H, *J* = 7.2 Hz, benzene proton), 7.54-7.56 (m, 4H, benzene and quinoline protons), 7.69-7.73 (m, 3H, benzene and quinoline protons), 7.89-7.92 (m, 2H, benzene and quinoline protons), 8.39 (d, 1H, *J* = 8.8 Hz, quinoline proton), 8.49 (d, 1H, *J* = 4.0 Hz, quinoline proton), 9.00 (s, 1H, CON*H*), 9.11 (bs, 2H, N*H*) ppm.

*Ethyl 2-(3-(quinolin-4-ylamino)phenyl)acetate (**6**)*. Mp: 159-161 °C; recrystallization solvent: acetonitrile; yield: 70%; ^{1}H NMR ($CDCl_3$ 400 MHz, δ; ppm) δ 1.29 (t, 3H, *J* = 7.2 Hz, $-COCH_2CH_3$), 3.65 (s, 2H, CH_2), 4.13 (q, 2H, *J* = 7.6 Hz, $-COCH_2CH_3$), 6.69 (d, 1H, *J* = 6.0 Hz, benzene proton), 7.23 (d, 1H, *J* = 6.8 Hz, benzene proton), 7.37-7.54 (m, 4H, benzene and quinoline protons), 7.65 (t, 1H, *J* = 7.6 Hz, quinoline proton), 8.00 (s, 1H, quinoline proton), 8.15 (d, 1H, *J* = 8.0 Hz, quinoline proton), 8.95 (d, 1H, *J* = 6.8 Hz, quinoline proton), 10.64 (bs, 1H, N*H*), 14.81 (bs, 1H, quinoline hydrochloride proton) ppm.

N-*(3-Nitrophenyl)quinolin-4-amine (**15**)*. Mp: 258-260 °C; recrystallization solvent: methanol; yield: 90%. ^{1}H NMR (DMSO, 400 MHz) δ 7.07 (d, 1H, *J* = 6.8 Hz, quinoline proton), 7.86 (t, 2H, *J* = 4.0 Hz, benzene protons), 8.00 (d, 1H, *J* = 8.4 Hz, benzene proton), 8.04-8.14 (m, 2H, benzene and quinoline protons), 8.23-8.25 (m, 1H, quinoline proton), 8.36-8.37 (m, 1H, quinoline proton), 8.63 (d, 1H, *J* = 6.8 Hz, quinoline proton), 8.80 (d, 1H, *J* = 8.4 Hz, quinoline proton), 11.08 (bs, 1H, NH) ppm.

*Ethyl 3-((2-amino-6-methylpyrimidin-4-yl)amino)benzoate (**17**).* Mp: 195-197 °C; recrystallization solvent: acetonitrile/methanol; yield: 70%. ^{1}H NMR (DMSO, 400 MHz) δ 1.34 (t, 3H, CH_2CH_3), 2.30 (s, 3H, CH_3), 4.35 (q, 2H, CH_2CH_3), 6.21 (s, 1H, pyrimidine proton), 7.52-7.56 (m, 1H, benzene proton), 7.73-7.75 (m, 1H, benzene proton), 8.06 (s, 1H, benzene proton), 8.35 (s, 1H, benzene proton), 10.85 (bs, 1H, NH), 12.92 (bs, 1H, pyrimidine hydrochloride proton) ppm.

*(3-(Quinolin-4-ylamino)phenyl)methanol (**19**).* Mp: 211-213 °C; recrystallization solvent: acetonitrile/methanol; yield: 68%. ^{1}H NMR (DMSO, 400 MHz) δ 4.51 (m, 2H, CH_2), 5.35 (bs, 1H, OH), 6.90-6.93 (m, 1H, benzene proton), 7.05-7.09 (m, 1H, benzene proton), 7.20-7.25 (m, 1H, benzene proton), 7.33-7.37 (m, 2H, benzene and quinoline protons), 7.49-7.53 (m, 1H, quinoline proton), 7.63-7.69 (m, 1H, quinoline proton), 7.81-7.87 (m, 1H, quinoline proton), 8.37-8.44 (m, 2H, quinoline protons), 8.96 (bs, 1H, NH) ppm.

Synthesis of 6-Methyl-*N*^{4}-(3-((3-(quinolin-4-ylamino)benzyl)amino)phenyl)pyrimidine-2,4-diamine (1f). The aldehyde **20** (0.81 mmol, 0.20 g) and the amine **9**[1] (0.80 mmol, 0.17 g) were stirred in anhydrous dichloroethane (5 mL) for 5 min. Afterwards, sodium triacetoxyborohydride (0.70 mmol, 0.22 g) was added and the resulting mixture was refluxed for 10 h. The reaction was quenched with 10 mL of water and extracted with dichloromethane (3 × 20 mL). The organic layer was washed with saturated sodium chloride (20 mL) and dried with sodium sulfate. Upon evaporation of the solvent, the crude product was purified by column chromatography on silica gel eluting with ethyl acetate/methanol 5:1 giving the pure compound **1f**. Mp: 98-100 °C; recrystallization solvent: toluene; yield: 50%. ^{1}H NMR (DMSO, 400 MHz) δ 2.04 (s, 3H, CH_3), 4.31 (d, 2H, J = 5.2 Hz, CH_2), 5.84 (s, 1H, pyrimidine proton), 5.96 (bs, 2H, NH_2), 6.18-6.34 (m, 2H, benzene protons), 6.85-6.88 (m, 2H, benzene protons), 6.92-6.94 (d, 2H, benzene and quinoline proton), 7.14 (d, 1H, J = 6.8 Hz, benzene proton), 7.20 (d, 1H, J = 8.0 Hz, benzene proton), 7.35-7.38 (m, 2H, benzene proton and N*H*), 7.52 (t,

1H, *J* = 7.6 Hz, quinoline proton), 7.68 (t, 1H, *J* = 8.0 Hz, quinoline proton), 7.85 (d, 1H, *J* = 8.4 Hz, quinoline proton), 8.34-8.38 (m, 2H, quinoline protons), 8.70 (bs, 1H, N*H*), 8.95 (bs, 1H, N*H*) ppm.

General Procedure for the Synthesis of 4a-c.

Triethylamine (2.21 mmol) and benzylchloroformate (1.48 mmol) were added to a solution of the appropriate *N*-(3- or 4-aminophenyl)-3- or 4-(quinolin-4-ylamino)benzamide (**25** for **4a**, **26** for **4b**, and **27** for **4c**) (0.21 mmol) in anhydrous THF (2 mL). The mixture was left under stirring for 2 h at room temperature and then was quenched by the addition of water (20 mL). The mixture was subsequently extracted with dichloromethane (3 × 20 mL) and washed with saturated sodium chloride solution (3 × 20 mL). The organic layer was dried over sodium sulphate, filtered and concentrated in vacuo. The crude product has been purified by column chromatography on silica gel eluting with ethyl acetate/methanol 50:1 giving the desired pure compound.

*Benzyl (4-(3-(quinolin-4-ylamino)benzamido)phenyl)carbamate (**4a**).* Mp: 262-264 °C; recrystallization solvent: methanol; yield: 75%. ^{1}H NMR (DMSO, 400 MHz) δ 5.15 (s, 2H, CH_2), 7.04 (d, 1H, *J* = 4.8 Hz, quinoline proton), 7.35-7.49 (m, 7H, benzene protons), 7.53-7.61 (m, 3H, benzene protons), 7.66-7.77 (m, 4H, benzene and quinoline protons), 7.82-7.94 (m, 2H, benzene and quinoline protons), 8.40 (d, 1H, *J* = 8.4 Hz, quinoline proton), 8.51 (d, 1H, *J* = 8.4 Hz, quinoline proton), 9.12 (bs, 1H, N*H*), 9.76 (bs, 1H, CON*H*), 10.22 (bs, 1H, CON*H*) ppm.

*Benzyl (3-(3-(quinolin-4-ylamino)benzamido)phenyl)carbamate (**4b**).* Mp: 250-252 °C; recrystallization solvent: methanol; yield: 43%. ^{1}H NMR (DMSO, 400 MHz) δ 5.16 (s, 2H, CH_2), 7.06 (d, 1H, *J* = 10.4 Hz, quinoline proton), 7.14-7.26 (m, 2H, benzene and quinoline protons), 7.31-7.45 (m, 6H, benzene and quinoline protons), 7.55-7.58 (m, 3H, benzene and quinoline protons), 7.66-7.74 (m, 2H, benzene and quinoline protons), 7.90-7.95 (m, 2H, benzene protons) , 8.02 (s, 1H, benzene proton), 8.42 (d, 1H, *J* = 8.4 Hz, quinoline proton), 8.52 (d, 1H, *J* = 8.4 Hz, quinoline proton), 9.14 (bs, 1H, N*H*), 9.80 (bs, 1H, CON*H*), 10.28 (bs, 1H, CON*H*) ppm.

*Benzyl (3-(4-(quinolin-4-ylamino)benzamido)phenyl)carbamate (**4c**).* Mp: 220-222 °C; recrystallization solvent: acetonitrile/methanol; yield: 27%. ^{1}H NMR (DMSO, 400 MHz) δ 5.17 (s, 2H, C*H*$_2$), 6.99 (d, 1H, *J* = 10.4 Hz, quinoline proton), 7.14-7.58 (m, 11H, benzene and quinoline protons), 7.77-7.81 (m, 1H, quinoline proton), 7.87-8.11 (m, 4H, benzene and quinoline protons), 8.39 (d, 1H, *J* = 8.4 Hz, quinoline proton), 8.58 (d, 1H, *J* = 8.4 Hz, quinoline proton), 9.30 (bs, 1H, N*H*), 9.87 (bs, 1H, CON*H*), 10.17 (bs, 1H, CON*H*) ppm.

Synthesis of the Intermediate Ethyl 3-((Quinolin-4-ylamino)methyl)benzoate (5). 4-Chloroquinoline (1.50 mmol, 0.25 g), ethyl 3-(aminomethyl)benzoate (0.74 mmol, 0.15 g) and sodium acetate (4.10 mmol, 0.33 g) were refluxed in distilled water for 5 h. After cooling, the reaction was extracted with ethyl acetate (3 × 15 mL). The organic layer was washed with saturated sodium chloride solution (15 mL), dried over sodium sulphate, filtered and concentrated in vacuo. The crude residue has been purified by column chromatography on silica gel eluting with ethyl acetate/methanol 15:1 obtaining the pure compound **5**. Mp: 188-190 °C; recrystallization solvent: acetonitrile/methanol; yield: 25%. ^{1}H NMR (DMSO, 400 MHz) δ 1.29 (t, 3H, *J* = 7.2 Hz, $COCH_2CH_3$), 4.13 (m, 4H, *J* = 7.6 Hz, $COCH_2CH_3$ and CH_2), 6.68 (d, 1H, *J* = 6.0 Hz, benzene proton), 7.25 (d, 1H, *J* = 6.8 Hz, benzene proton), 7.34 - 7.51 (m, 4H, benzene and quinoline protons), 7.62 (t, 1H, *J* = 7.6 Hz, quinoline proton), 8.01 (s, 1H, quinoline proton), 8.13 (d, 1H, *J* = 8.0 Hz, quinoline proton), 8.93 (d, 1H, *J* = 6.8 Hz, quinoline proton), 10.62 (bs, 1H, NH) ppm.

General Procedure for the Synthesis of Intermediate Compounds 7, 8, and 18.

A solution of the appropriate ethyl ester (**5** for **7**, **6** for **8**, and **17** for **18**) (2.98 mmol) and 2 N potassium hydroxyde (11.92 mmol) in ethanol/water mixture (10 mL, 1:1) was stirred overnight at room temperature. Subsequently, most of the ethanol was distilled out and pH was adjusted to 5 *via* the slow addition of 2 N hydrochloric acid at 0 °C. The precipitated acid was filtered and washed with diethyl ether, yielding the desired pure acidic compound as hydrochloride salt.

*3-((Quinolin-4-ylamino)methyl)benzoic acid (**7**).* Mp: 180-182 °C; recrystallization solvent: acetonitrile/methanol; yield: 30%. ^{1}H NMR (DMSO-d_6, 400 MHz) δ 4.87 (s, 2H, CH_2), 6.77 (d, 1H, *J* = 4.4 Hz, quinoline proton), 7.50-7.52 (m, 1H, benzene proton), 7.68-7.75 (m, 2H, quinoline and benzene protons), 7.87-8.01 (m, 4H, quinoline and benzene protons), 8.39 (m, 1H, quinoline proton), 8.51 (d, 1H, *J* = 6.8 Hz, quinoline proton), 9.86 (bs, 1H, NH) 14.61 (bs, 1H, COOH), 14.81 (bs, 1H, quinoline hydrochloride proton) ppm.

*2-(3-(Quinolin-4-ylamino)phenyl)acetic acid (**8**).* Mp: 116-118 °C; recrystallization solvent: toluene; yield: 91%. ^{1}H NMR (DMSO, 400 MHz) δ 3.70 (s, 2H, CH_2), 6.79-6.82 (m, 1H, benzene proton), 7.29-7.41 (m, 3H, benzene protons), 7.49-7.54 (m, 1H, quinoline proton), 7.79-7.84 (m, 1H, quinoline proton), 8.01-8.08 (m, 2H, quinoline protons), 8.50-8.53 (m, 1H, quinoline proton), 8.77-8.79 (m, 1H, quinoline proton), 10.93 (s, 1H, NH), 12.49 (bs, 1H, COOH), 14.57 (bs, 1H, quinoline hydrochloride proton) ppm.

*3-((2-Amino-6-methylpyrimidin-4-yl)amino)benzoic acid (**18**).* Mp: >300 °C; recrystallization solvent: methanol; yield: 90%. ^{1}H NMR (DMSO, 400 MHz) δ 2.29 (s, 3H, CH_3), 6.19 (s, 1H, pyrimidine proton), 7.49-7.55 (m, 1H, benzene proton), 7.71-7.78 (m, 1H, benzene proton), 8.06 (s, 1H, benzene proton), 8.24 (s, 1H, benzene proton), 10.74 (bs, 1H, N*H*,), 12.92 (bs, 1H, pyrimidine hydrochloride proton) ppm.

Synthesis of the Intermediate 6-Methyl-N^4-(3-nitrobenzyl)pyrimidine-2,4-diamine 12.

2-Amino-4-chloro-6-methylpyrimidine (1.74 mmol, 0.25 g), 3-nitrobenzylamine (3.48 mmol, 0.53 g) and *N,N*-diisopropylethylamine (DIPEA) (4.33 mmol, 0.56 g) were dissolved in 6 mL of DMSO. The reaction was carried out using microwave (Biotage initiator) at 160 °C for 45 min. The reaction mixture was quenched with water (10 mL), extracted with ethyl acetate (3 × 10 mL) and washed with saturated sodium chloride solution (15 mL). The organic layer was dried over sodium sulphate, filtered and concentrated in vacuo. The crude residue has been purified by column chromatography on silica gel

eluting with chloroform/methanol 8:1 obtaining the pure compound **12**. Mp: 63-65 °C; recrystallization solvent: cyclohexane; yield: 80%. ^{1}H NMR ($CDCl_3$, 400 MHz) δ 2.20 (s, 3H, CH_3), 4.95 (s, 2H, *J* = 5.6 Hz, CH_2), 4.93 (bs, 2H, NH_2), 5.69 (s, 1H, pyrimidine proton), 7.52 (t, 1H, *J* = 8.0 Hz, benzene proton), 7.67 (d, 1H, *J* = 7.2 Hz, benzene proton), 8.14 (d, 1H, *J* = 8.0 Hz, benzene proton), 8.19 (s, 1H, benzene proton) ppm.

General Procedure for the Synthesis of Anilines 13, 14, and 16.

Two drops of a 37% hydrochloric acid solution were slowly added at 0 °C to a solution of the appropriate nitroderivative (**11** for **13**, **12** for **14**, and **15** for **16**) (0.67 mmol) and stannous chloride dihydrate (3.32 mmol) in ethanol (5 mL). The reaction was refluxed for 5 h. Afterwards, most of the ethanol was distilled out and 2 N sodium carbonate solution (20 mL) was added to the mixture followed by extraction with ethyl acetate (3 × 30 mL). The collected organic layers were washed with brine (3 × 30 mL), dried over sodium sulphate, filtered and concentrated in vacuo. The crude solid was subsequently triturated with diethyl ether and filtered again, giving the desired pure compound.

*N-(3-Aminobenzyl)-3-(quinolin-4-ylamino)benzamide (**13**)*. Mp: 213-215 °C; recrystallization solvent: acetonitrile/methanol; yield: 62%. ^{1}H NMR (DMSO, 400 MHz) δ 4.34 (d, 2H, *J* = 4.4 Hz, CH_2), 5.02 (s, 2H, NH_2), 6.44-6.56 (m, 3H, benzene protons), 6.95-7.02 (m, 2H, benzene protons), 7.48-7.58 (m, 3H, benzene and quinoline protons), 7.66-7.73 (m, 2H, benzene and quinoline protons), 7.89-7.91 (m, 2H, benzene and quinoline protons), 8.39 (d, 1H, *J* = 8.8 Hz, quinoline proton), 8.49 (d, 1H, *J* = 4.0 Hz, quinoline proton), 8.96 (bs, 1H, CONH), 9.08 (bs, 1H, NH) ppm.

*N^4-(3-Aminobenzyl)-6-methylpyrimidine-2,4-diamine (**14**)*. Mp: 68-70 °C; recrystallization solvent: cyclohexane; yield: 87%. ^{1}H NMR (DMSO-*d* $_6$, 400 MHz) δ 2.14 (s, 3H, CH_3), 3.91 (bs, 2H, NH_2), 5.18 (bs, 2H, NH_2), 5.60 (s, 1H, pyrimidine proton), 6.57-6.66 (m, 3H, benzene protons), 7.08-7.11 (m, 1H, benzene proton), 8.64 (bs, 1H, NH) ppm.

*N^1-(Quinolin-4-yl)benzene-1,3-diamine (**16**).* Mp: 106-109 °C; recrystallization solvent: toluene; yield: 92%. ^{1}H NMR (DMSO, 400 MHz) δ 5.18 (bs, 2H, NH_2), 6.37 (d, 1H, benzene proton), 6.50 (d, 1H, benzene proton), 6.60 (s, 1H, benzene proton), 6.91 (d, 1H, *J* = 6.8 Hz, benzene proton), 7.05 (t, 1H, *J* = 8.0 Hz, quinoline proton), 7.49 (t, 1H, *J* = 7.6 Hz, quinoline proton), 7.65 (t, 1H, *J* = 8.0 Hz, quinoline proton), 7.85 (d, 1H, *J* = 8.4 Hz, quinoline proton), 8.35-8.43 (m, 2H, quinoline protons), 8.73 (bs, 1H, NH) ppm.

Synthesis of the Intermediate 3-(Quinolin-4-ylamino)benzaldehyde (20). Manganese dioxide (2.10 mmol, 0.18 g) was added to a solution of (3-(quinolin-4-ylamino)phenyl)methanol **19** (0.42 mmol, 0.10 g) in anhydrous THF (2 mL), and the mixture was stirred overnight at 60 °C. The mixture was filtered through a pad of celite and concentrated in vacuo. The crude residue has been purified by column chromatography on silica gel eluting with ethyl acetate obtaining the pure compound **20**.

Mp: 165-167 °C; recrystallization solvent: acetonitrile; yield: 48%. ^{1}H NMR (DMSO, 400 MHz) δ 6.81 (d, 1H, *J* = 6.4 Hz, benzene proton), 7.35-7.47 (m, 3H, benzene protons), 7.54 (t, 1H, *J* = 8.0 Hz, quinoline proton), 7.82 (t, 1H, *J* = 7.8 Hz, quinoline proton), 8.04-8.15 (m, 3H, NH -and benzene protons), 8.52 (d, 1H, *J* = 7.6 Hz, quinoline proton), 8.79 (d, 1H, *J* = 8.4 Hz, quinoline proton), 9.88 (s, 1H, CHO proton) ppm.

Table S1. Elemental analysis of the final compounds **1a-f**, **2a-c**, **3a-c**, and **4a-c**.

compd	MW	%, calculated			%, found		
		C	H	N	C	H	N
**1a**	475.56	70.72	5.30	20.62	70.59	5.25	20.84
**1b**	475.56	70.72	5.30	20.62	70.81	5.42	20.45
**1c**	475.56	70.72	5.30	20.62	70.95	5.48	20.39
**1d**	475.56	70.72	5.30	20.62	70.54	5.22	20.76
**1e**	461.53	70.27	5.02	21.24	70.45	5.14	21.02
**1f**	447.55	72.46	5.63		72.24	5.46	
**2a**	481.56	77.32	4.81	14.54	77.18	4.68	14.79
**2b**	481.56	77.32	4.81	14.54	77.45	4.87	14.38
**2c**	481.56	77.32	4.81	14.54	77.26	4.77	14.63
**3a**	441.50	62.57	5.25	28.55	62.33	5.08	28.81
**3b**	441.50	62.57	5.25	28.55	62.81	5.40	28.28
**3c**	441.50	62.57	5.25	28.55	62.44	5.16	28.77
**4a**	488.55	73.76	4.95	11.47	73.90	5.09	11.22
**4b**	488.55	73.76	4.95	11.47	73.48	4.79	11.63
**4c**	488.55	73.76	4.95	11.47	73.81	5.11	11.19

Experimental procedure for Fluorescence Resonance Energy Transfer (FRET) melting assay

Oligonucleotides labeled at 5’ with FAM (6-carboxyfluorescein) as donor fluorophore and TAMRA (6-carboxytetramethylrhodamine) at 3’ as the acceptor fluorophore were purchased from STAB VIDA (Portugal). Each oligonucleotide was initially diluted to 100 μM in water (Molecular Biology Reagent, Sigma). Stock solutions of 20 μM and subsequent dilutions were made with FRET buffer (60 mM KCl, potassium cacodylate, pH 7.4). Tagged oligonucleotides at 0.4 μM were annealed by heating at 90-95 °C for 10 min, followed by slow cooling to room temperature. Stock solutions of compounds (1 mM) were prepared in 10% DMSO. Subsequent dilutions were performed using FRET buffer. Annealed DNA (50 μL) and test compound solutions (50 μL) were distributed across 96-well RT-PCR plates (PCR-96-FLT-C, Axygen, Inc). Fluorescence readings (performed in a 7300 RT-PCR equipment from Applied Biosystems) were taken at intervals of 0.5 °C in the range 31–95 °C, with the temperature being maintained for 30 seconds prior to each reading. Experiments were performed in triplicate. Final analysis of the data was carried out with GraphPad Prism v.5.0 (GraphPad Software Inc., La Jolla, CA, USA). The advanced curve-fitting function in GraphPad Prism (nonlinear regression fit) was used for calculation of ΔT_m values. Only results with fitting r^2 values > 0.75 (std error < 0.25) were considered.

Table S2. Synthetic oligonucleotides used in FRET experiments.

Code	Sequence	Tm	Topology
KRAS21R	5’-FAM-AGG GCG GTG TGG GAA GAG GGA-TAMRA-3’	52 °C	Parallel G4
Telo21	5’-FAM-GGG TTA GGG TTA GGG TTA GGG-TAMRA-3’	57 °C	Hybrid G4

Table S3. ΔTm values for F21T and KRAS stabilized by DNMT inhibitors at 5 µM and 10 µM. Py4P used as positive control at 1 µM.

Comp	F21T ΔTm (ºC) (5 µM)	F21T ΔTm (ºC) (10 µM)	KRAS ΔTm (ºC) (5 µM)	KRAS ΔTm (ºC) (10 µM)
SGI-1027	1.9	7.2	6.7	8.7
**1**	8.0	8.3	5.7	12.8
**2**	0.6	6.2	1.0	9.6
**2a**	0	5.1	0	6.4
**2b**	2.8	5.0	1.0	7.7
**2c**	1	1.2	0	3.4
**4**	2.7	6.9	1	10.4
**4a**	0	4.3	2.5	4.6
**4b**	3.8	1.5	2.8	2.5
**4c**	0.8	1.5	1.0	2.6
Py4P		21.2	18.8	

Experimental procedure of kinase inhibitory assays

The test compound, reference compound or water (control) were mixed with the enzyme (for the exact amount see table below) in the appropriate buffer. Thereafter, the reaction was initiated by adding the required amount of the appropriate substrate and of ATP, and the mixture is incubated at room temperature (variable reaction time, see table below). For control basal measurements, the enzyme was omitted from the reaction mixture. Following incubation, the reaction was stopped by adding 13 mM EDTA. After 5 min, the proper antibody labelled with europium chelate was added. After 60 more min, the fluorescence transfer was measured at λ_{ex}=337 nm, λ_{em}=620 nm and λ_{em}=665 nm using a microplate reader (Envision, Perkin Elmer). The enzyme activity was determined by dividing the signal measured at 665 nm by that measured at 620 nm (ratio). The results are expressed as a percent inhibition of the control enzyme activity. The standard inhibitory reference compound is staurosporine when not differently indicated (see table below), which has been tested in each experiment at several concentrations to obtain an inhibition curve from which its IC_{50} value is calculated.

<u>General information</u>

- Assay volume and format: 10 μL in 384-well plate
- Compound addition: [100x] solution in solvent then [5x] solution in water
- Maximum tolerable DMSO concentration: 1%

Table S4. Screening of **2a** on a panel of kinases. Percentage of inhibition at 10 µM.

kinases	% inhibition at 10 µM by 2a	Enzyme quantity (ng)[a]	buffer[b]	substrate	[s][c]	[ATP]	Incubation time	Antibody	Ref. comp.[d]	Ref. (lit)
Abl kinase (h)	0	0.4	A	Ulight-TK peptide	100 nM	10 µM	30 min	anti-phospho-PT66		[2]
Akt1/PKBalpha (h)	-178	1.2	B	Ulight-CREBtide (CKRREILSRRPSYRK)	25 nM	30 µM	60 min	anti-phospho-CREB		[3]
AurA/Aur2 kinase (h)	-20	2.36	B	Ulight-RRRSLLE (PLK)	100 nM	10 µM	15 min	anti-phospho-PLK		[4]
CaMK2alpha (h)	-99	8.1	C	Ulight-CGSGSGRPRTSSFAEG (Crosstide)	50 nM	10 µM	30 min	anti-phospho-Crosstide	AIP	[5]
CDC2/CDK1 (h) (cycB)	16	3	B	Ulight-CFFKNIVTPRTPPPSQGK-amide (MBP)	100 nM	10 µM	15 min	anti-phospho-MBP		[6]
CHK1 (h)	16	0.6	B	Ulight-CREBtide (CKRREILSRRPSYRK)	25 nM	30 µM	30 min	anti-phospho-CREB		[7]
CHK2 (h)	-195	1.32	B	Ulight-CREBtide (CKRREILSRRPSYRK)	25 nM	30 µM	15 min	anti-phospho-CREB		[8]
c-Met kinase (h)	-54	0.4	B + 300 nM poly-D-Lys	Ulight-CAGAGAIETDKEYYTVKD (JAK1)	25 nM	10 µM	60 min	anti-phospho-PT66		[9]
EGFR kinase (h)	36	0.0452	A + 100 nM poly-D-Lys	Ulight-CAGAGAIETDKEYYTVKD (JAK1)	100 nM	10 µM	15 min	anti-phospho-PT66	PD153035	[10]
EphA2 kinase (h)	-184	0.2	A	Ulight-TK peptide	50 nM	10 µM	30 min	anti-phospho-PT66		[11]
EphA3 kinase (h)	-57	0.56	A	Ulight-TK peptide	50 nM	30 µM	60 min	anti-phospho-PT66		[12]
EphB4 kinase (h)	-375	0.05	B	Ulight-TK peptide	100 nM	50 µM	90 min	anti-phospho-PT66		[13]
ERK2 (h) (P42mapk)	9	1.38	B	Ulight-CFFKNIVTPRTPPPSQGK-amide (MBP)	100 nM	10 µM	15 min	anti-phospho-MBP		[14]
FGFR1 kinase (h)	-220	0.252	B	Ulight-CAGAGAIETDKEYYTVKD (JAK1)	100 nM	100 µM	60 min	anti-phospho-PT66		[15]
FGFR2 kinase (h)	1	0.0075	B + 50 nM poly-D-Lys	Ulight-CAGAGAIETDKEYYTVKD (JAK1)	25 nM	10 µM	15 min	anti-phospho-PT66		[16]
FGFR3 kinase (h)	-272	0.7	A	Ulight-CAGAGAIETDKEYYTVKD (JAK1)	100 nM	10 µM	90 min	anti-phospho-PT66		[17]
GSK3beta (h)	-72	21.9	B	Ulight-CFFKNIVTPRTPPPSQGK-amide (MBP)	100 nM	10 µM	90 min	anti-phospho-MBP		[18]
HGK (h) (MAP4K4)	-247	19.5	B	Ulight-FLGFTYVAP (P70S6K)	50 nM	1 µM	90 min	anti-phospho-P70S6K		[19]
IKKalpha (h)	70	11.2	B	Ulight-IkappaB-alpha	100 nM	5 µM	30 min	anti-phospho-IkappaB-alpha		[20]

IRAK4 (h)	-214	16.72	B	Ulight-FLGFTYVAP (P70S6K)	100 nM	2.5 µM	90 min	anti-phospho-P70S6K		[21]
IRK (h) (InsR)	86	0.0156	B	Ulight-Poly GAT[EAY(1:1:1)]n	50 nM	30 µM	10 min	anti-phospho-PT66		[22]
JAK3 (h)	-100	0.204	B	Ulight-CAGAGAIETDKE YYTVKD (JAK1)	100 nM	0.5 µM	60 min	anti-phospho-PT66		[23]
JNK1 (h)	-91	6.8	B	Ulight-CFFKNIVTPRTPP PSQGK-amide (MBP)	100 nM	10 µM	60 min	anti-phospho-MBP		[24]
KDR kinase (h) (VEGFR2)	-281	0.88	B	Ulight-CAGAGAIETDKE YYTVKD (JAK1)	100 nM	25 µM	60 min	anti-phospho-PT66		[25]
Lck kinase (h)	23	1	B	Ulight-Poly GAT[EAY(1:1:1)]n	25 nM	10 µM	10 min	anti-phospho-PT66		[2]
MAPKAPK2 (h)	-1011	2.44	B	Ulight-CREBtide (CKRREILSRRPS YRK)	25 nM	1 µM	15 min	anti-phospho-CREB		[26]
MARK1 (h)	-209	11.6	B	Ulight-RRRSLLE (PLK)	50 nM	1 µM	30 min	anti-phospho-PLK		[27]
MNK2 (h)	-6	5	B	Ulight-CREBtide (CKRREILSRRPS YRK)	25 nM	100 µM	90 min	anti-phospho-CREB		[28]
MST4 kinase (h)	-567	5.2	B	Ulight-CRFARKGSLRQK NV (PKC)	50 nM	10 µM	30 min	anti-phophohist one H3		[29]
NEK2 (h)	-72	2.728	B	Ulight-FLGFTYVAP (P70S6K)	50 nM	10 µM	60 min	anti-phospho-P70S6K		[30]
p38alpha kinase (h)	-64	6	B	Ulight-CFFKNIVTPRTPP PSQGK-amide (MBP)	100 nM	100 µM	30 min	anti-phospho-MBP	SB20219 0	[31]
PAK2 (h)	-150	17.6	B	Ulight-RRRSLLE (PLK)	50 nM	50 µM	60 min	anti-phospho-PLK		[32]
PAK4 (h)	-17	20	B	Ulight-RRRSLLE (PLK)	50 nM	1 µM	30 min	anti-phospho-PLK		[33]
PDK1 (h)	-169	50	B	Ulight-FLGFTYVAP (P70S6K)	400 nM	10 µM	90 min	anti-phospho-P70S6K		[34]
Pim2 kinase (h)	-51	6.36	B	Ulight-CREBtide (CKRREILSRRPS YRK)	25 nM	3 µM	60 min	anti-phospho-CREB		[35]
PKA (h)	26	0.005	A	Ulight-PLK (Ser 137)	50 nM	1 µM	10 min	anti-phospho-PLK		[36]
PKCbeta 2 (h)	79	0.06	B	Ulight-CREBtide (CKRREILSRRPS YRK)	25 nM	30 µM	15 min	anti-phospho-CREB		[37]
PLK1 (h)	-214	9.6	B	Ulight-FLGFTYVAP (P70S6K)	40 nM	5 µM	60 min	anti-phospho-P70S6K		[38]
RAF-1 kinase (h)	79	5	B	Ulight-ARTKQTARKSTG GKAPRKQLAGC G (histone H3)	50 nM	10 µM	180 min	anti-phospho-histone H3		[39]
ROCK1 (h)	-61	8.2	B	Ulight-RRRSLLE (PLK)	50 nM	1 µM	30 min	anti-phospho-PLK		[40]
SGK1 (h)	28	3.45	A	Ulight-RRRSLLE (PLK)	50 nM	10 µM	30 min	anti-phospho-PLK		[41]

SIK (h)	22	9	B	Ulight-CREBtide (CKRREILSRRPS YRK)	25 nM	30 µM	90 min	anti-phospho-CREB	Ro-318220	[42]
Src kinase (h)	94	0.06	B	Ulight-Poly GAT[EAY(1:1:1)]n	5 nM	5 µM	10 min	anti-phospho-PT66		[43]
TAOK2 (TAO1) (h)	-203	12.8	B	Ulight-FLGFTYVAP (P70S6K)	40 nM	5 µM	60 min	anti-phospho-P70S6K		[44]
TRKA (h)	98	0.86	B	Ulight-Poly GAT[EAY(1:1:1)]n	5 nM	100 µM	10 min	anti-phospho-PT66		[45]

[a]Amount of enzyme (expressed in nanograms) in 10 µL reaction volume; [b]Buffer composition: A: 40 mM Hepes/ Tris (pH 7.4), 0.8 mM EGTA/Tris, 8 mM $MgCl_2$, 3.6 mM DTT, 0.008% Tween 20; B: 40 mM Hepes/ Tris (pH 7.4), 0.8 mM EGTA/Tris, 8 mM $MgCl_2$, 1.6 mM DTT, 0.008% Tween 20; C: 40 mM Hepes/Tris (pH 7.4), 0.8 mM EGTA/Tris, 8 mM $MgCl_2$, 2.5 mM $CaCl_2$, 1.6 mM DTT, 0.008% Tween 20, and 5 µg/ml calmodulin; [c]substrate concentration; [d]Only when different from staurosporine.

Whole original Western Blots

Experiment 1: dose-dependent protein expression
2b (samples 1 and 2) and 4c (samples 5 and 6) were used a 0.1 and 1 μM and protein levels were analysed respect to the ctr (DMSO, samples 3 and 4)

GAPDH

38 KDa

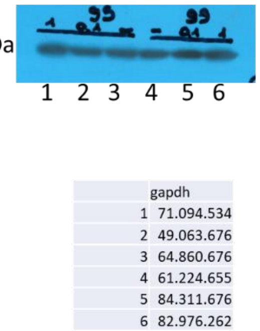

1 2 3 4 5 6

DNMT1

180 KDa

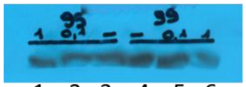

1 2 3 4 5 6

DNMT3A

130 KDa

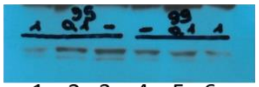

1 2 3 4 5 6

	gapdh
1	71.094.534
2	49.063.676
3	64.860.676
4	61.224.655
5	84.311.676
6	82.976.262

	dnmt1	dnmt1/gapdh	fold
1	30.296.463	0,426143352	0,425624
2	39.501.525	0,805107326	0,804126
3	64.939.798	1,001219876	1
4	78.359.978	1,279876187	1
5	83.449.869	0,98977832	0,773339
6	32.034.848	0,386072441	0,301648

	dnmt3a	dnmt3a/gapdh	fold
1	13.167.430	0,18521016	0,256815
2	35.673.563	0,727087041	1,008187
3	46.776.392	0,721182616	1
4	29.251.472	0,477772753	1
5	25.631.108	0,304004252	0,636295
6	8.868.007	0,106874024	0,223692

Experiment 2: dose-dependent protein expression
2b (samples 1 and 2) and 4c (samples 5 and 6) were used a 0.1 and 1 μM and protein levels were analysed respect to the ctr (DMSO, samples 3 and 4)

GAPDH

38 KDa

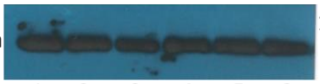

1 2 3 4 5 6

DNMT1

180 KDa

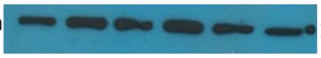

1 2 3 4 5 6

DNMT3A

130 KDa

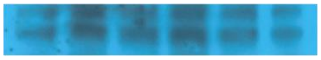

1 2 3 4 5 6

	gapdh
1	52.762.945
2	46.052.581
3	40.664.045
4	41.230.075
5	36.555.288
6	31.596.359

	dnmt1	dnmt1/gapdh	fold
1	73.466.588	1,392389821	0,687809
2	95.524.616	2,074251078	1,024633
3	82.319.659	2,024384416	1
4	117.519.940	2,850345045	1
5	93.877.152	2,568086777	0,900974
6	51.362.058	1,625568881	0,570306

	dnmt3a	dnmt3a/gapdh	fold
1	34.704.165	0,657737443	0,446652
2	76.574.316	1,662758407	1,129135
3	59.881.664	1,472594869	1
4	69.128.421	1,676650382	1
5	36.722.411	1,004571783	0,599154
6	18.469.198	0,584535653	0,348633

Experiment 3: protein expression after the treatment with 4c or the co-treatment with 4c and bortezomib to inhibit proteasome-dependent protein degradation. 1° of 2 independent biological replicates, both with 3 independent technical replicates.
4c (samples 1-3) and 4c+bortezomib (samples 4-6) were used a 1 µM an 10nM respectively. DMSO-treated sample (7) was used as a reference.

GAPDH

38 KDa

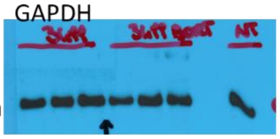

1 2 3 4 5 6 7

DNMT1

180 KDa

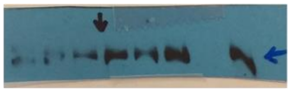

1 2 3 4 5 6 7

DNMT3A

130 KDa

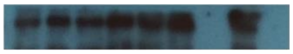

1 2 3 4 5 6 7

	gapdh
1	87.631.836
2	91.610.128
3	114.602.685
4	76.244.300
5	103.165.472
6	99.143.886
7	100.744.765

	dnmt1	dnmt1/gapdh
1	17.777.865	0,202869936
2	24.871.945	0,271497765
3	32.793.028	0,286145373
4	45.724.744	0,599713605
5	24.500.886	0,237491144
6	67.147.522	0,677273453
7	54.768.752	0,543638689

	dnmt3a	dnmt3a/gapdh
1	10.834.057	0,123631519
2	21.351.309	0,233067123
3	10.448.300	0,091169766
4	30.351.321	0,39807987
5	20.774.693	0,201372539
6	44.258.898	0,446410765
7	69.994.836	0,694773927

Experiment 4: protein expression after the treatment with 4c or the co-treatment with 4c and bortezomib to inhibit proteasome-dependent protein degradation. 2° of 2 independent biological replicates, both with 3 independent technical replicates.
4c (samples 1-3) and 4c+bortezomib (samples 4-6) were used a 1 µM an 10nM respectively. DMSO-treated sample (0) was used as a reference.

GAPDH

38 KDa

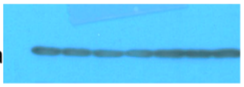

0 1 2 3 4 5 6

DNMT1

180 KDa

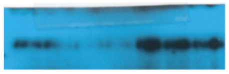

0 1 2 3 4 5 6

DNMT3A

130 KDa

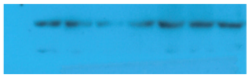

0 1 2 3 4 5 6

	gapdh
1	18.021.618
2	17.758.539
3	17.904.246
4	18.366.761
5	27.439.711
6	24.899.518
7	27.563.497

	dnmt1	dnmt1/gapdh
1	10.903.295	0,605012
2	9.729.477	0,547876
3	6.057.347	0,338319
4	6.550.799	0,356666
5	22.158.253	0,807525
6	22.203.124	0,891709
7	14.730.015	0,534403

	dnmt3a	dnmt3a/gapdh
1	18.269.326	1,013745
2	11.835.427	0,666464
3	5.712.278	0,319046
4	14.024.712	0,763592
5	25.391.035	0,925339
6	23.082.476	0,927025
7	17.084.324	0,619817

References

1. Valente S, Liu Y, Schnekenburger M, Zwergel C, Cosconati S, Gros C, Tardugno M, Labella D, Florean C, Minden S, et al: **Selective non-nucleoside inhibitors of human DNA methyltransferases active in cancer including in cancer stem cells.** *J Med Chem* 2014, **57:**701-713.
2. Park YW, Cummings RT, Wu L, Zheng S, Cameron PM, Woods A, Zaller DM, Marcy AI, Hermes JD: **Homogeneous proximity tyrosine kinase assays: scintillation proximity assay versus homogeneous time-resolved fluorescence.** *Anal Biochem* 1999, **269:**94-104.
3. Barnett SF, Defeo-Jones D, Fu S, Hancock PJ, Haskell KM, Jones RE, Kahana JA, Kral AM, Leander K, Lee LL, et al: **Identification and characterization of pleckstrin-homology-domain-dependent and isoenzyme-specific Akt inhibitors.** *Biochem J* 2005, **385:**399-408.
4. Sun C, Newbatt Y, Douglas L, Workman P, Aherne W, Linardopoulos S: **High-throughput screening assay for identification of small molecule inhibitors of Aurora2/STK15 kinase.** *J Biomol Screen* 2004, **9:**391-397.
5. Ishida A, Fujisawa H: **Stabilization of calmodulin-dependent protein kinase II through the autoinhibitory domain.** *J Biol Chem* 1995, **270:**2163-2170.
6. Meijer L, Borgne A, Mulner O, Chong JP, Blow JJ, Inagaki N, Inagaki M, Delcros JG, Moulinoux JP: **Biochemical and cellular effects of roscovitine, a potent and selective inhibitor of the cyclin-dependent kinases cdc2, cdk2 and cdk5.** *Eur J Biochem* 1997, **243:**527-536.
7. Zhao B, Bower MJ, McDevitt PJ, Zhao H, Davis ST, Johanson KO, Green SM, Concha NO, Zhou BB: **Structural basis for Chk1 inhibition by UCN-01.** *J Biol Chem* 2002, **277:**46609-46615.
8. Ng CP, Lee HC, Ho CW, Arooz T, Siu WY, Lau A, Poon RY: **Differential mode of regulation of the checkpoint kinases CHK1 and CHK2 by their regulatory domains.** *J Biol Chem* 2004, **279:**8808-8819.
9. Bardelli A, Longati P, Gramaglia D, Basilico C, Tamagnone L, Giordano S, Ballinari D, Michieli P, Comoglio PM: **Uncoupling signal transducers from oncogenic MET mutants abrogates cell transformation and inhibits invasive growth.** *Proc Natl Acad Sci U S A* 1998, **95:**14379-14383.
10. Weber W, Bertics PJ, Gill GN: **Immunoaffinity purification of the epidermal growth factor receptor. Stoichiometry of binding and kinetics of self-phosphorylation.** *J Biol Chem* 1984, **259:**14631-14636.
11. Kinch MS, Moore MB, Harpole DH, Jr.: **Predictive value of the EphA2 receptor tyrosine kinase in lung cancer recurrence and survival.** *Clin Cancer Res* 2003, **9:**613-618.
12. Sharfe N, Freywald A, Toro A, Roifman CM: **Ephrin-A1 induces c-Cbl phosphorylation and EphA receptor down-regulation in T cells.** *J Immunol* 2003, **170:**6024-6032.
13. Sturz A, Bader B, Thierauch KH, Glienke J: **EphB4 signaling is capable of mediating ephrinB2-induced inhibition of cell migration.** *Biochem Biophys Res Commun* 2004, **313:**80-88.
14. Bardwell AJ, Abdollahi M, Bardwell L: **Docking sites on mitogen-activated protein kinase (MAPK) kinases, MAPK phosphatases and the Elk-1 transcription factor compete for MAPK binding and are crucial for enzymic activity.** *Biochem J* 2003, **370:**1077-1085.
15. Mohammadi M, McMahon G, Sun L, Tang C, Hirth P, Yeh BK, Hubbard SR, Schlessinger J: **Structures of the tyrosine kinase domain of fibroblast growth factor receptor in complex with inhibitors.** *Science* 1997, **276:**955-960.
16. Robertson SC, Meyer AN, Hart KC, Galvin BD, Webster MK, Donoghue DJ: **Activating mutations in the extracellular domain of the fibroblast growth factor receptor 2 function by disruption of the disulfide bond in the third immunoglobulin-like domain.** *Proc Natl Acad Sci U S A* 1998, **95:**4567-4572.
17. Hart KC, Robertson SC, Kanemitsu MY, Meyer AN, Tynan JA, Donoghue DJ: **Transformation and Stat activation by derivatives of FGFR1, FGFR3, and FGFR4.** *Oncogene* 2000, **19:**3309-3320.

18. Meijer L, Skaltsounis AL, Magiatis P, Polychronopoulos P, Knockaert M, Leost M, Ryan XP, Vonica CA, Brivanlou A, Dajani R, et al: **GSK-3-selective inhibitors derived from Tyrian purple indirubins.** *Chem Biol* 2003, **10:**1255-1266.
19. Yao Z, Zhou G, Wang XS, Brown A, Diener K, Gan H, Tan TH: **A novel human STE20-related protein kinase, HGK, that specifically activates the c-Jun N-terminal kinase signaling pathway.** *J Biol Chem* 1999, **274:**2118-2125.
20. Huynh QK, Boddupalli H, Rouw SA, Koboldt CM, Hall T, Sommers C, Hauser SD, Pierce JL, Combs RG, Reitz BA, et al: **Characterization of the recombinant IKK1/IKK2 heterodimer. Mechanisms regulating kinase activity.** *J Biol Chem* 2000, **275:**25883-25891.
21. Li S, Strelow A, Fontana EJ, Wesche H: **IRAK-4: a novel member of the IRAK family with the properties of an IRAK-kinase.** *Proc Natl Acad Sci U S A* 2002, **99:**5567-5572.
22. al-Hasani H, Passlack W, Klein HW: **Phosphoryl exchange is involved in the mechanism of the insulin receptor kinase.** *FEBS Lett* 1994, **349:**17-22.
23. Zhou YJ, Hanson EP, Chen YQ, Magnuson K, Chen M, Swann PG, Wange RL, Changelian PS, O'Shea JJ: **Distinct tyrosine phosphorylation sites in JAK3 kinase domain positively and negatively regulate its enzymatic activity.** *Proc Natl Acad Sci U S A* 1997, **94:**13850-13855.
24. Bennett BL, Sasaki DT, Murray BW, O'Leary EC, Sakata ST, Xu W, Leisten JC, Motiwala A, Pierce S, Satoh Y, et al: **SP600125, an anthrapyrazolone inhibitor of Jun N-terminal kinase.** *Proc Natl Acad Sci U S A* 2001, **98:**13681-13686.
25. Itokawa T, Nokihara H, Nishioka Y, Sone S, Iwamoto Y, Yamada Y, Cherrington J, McMahon G, Shibuya M, Kuwano M, Ono M: **Antiangiogenic effect by SU5416 is partly attributable to inhibition of Flt-1 receptor signaling.** *Mol Cancer Ther* 2002, **1:**295-302.
26. Tan Y, Rouse J, Zhang A, Cariati S, Cohen P, Comb MJ: **FGF and stress regulate CREB and ATF-1 via a pathway involving p38 MAP kinase and MAPKAP kinase-2.** *EMBO J* 1996, **15:**4629-4642.
27. Timm T, Marx A, Panneerselvam S, Mandelkow E, Mandelkow EM: **Structure and regulation of MARK, a kinase involved in abnormal phosphorylation of Tau protein.** *BMC Neurosci* 2008, **9 Suppl 2:**S9.
28. Waskiewicz AJ, Flynn A, Proud CG, Cooper JA: **Mitogen-activated protein kinases activate the serine/threonine kinases Mnk1 and Mnk2.** *EMBO J* 1997, **16:**1909-1920.
29. Qian Z, Lin C, Espinosa R, LeBeau M, Rosner MR: **Cloning and characterization of MST4, a novel Ste20-like kinase.** *J Biol Chem* 2001, **276:**22439-22445.
30. Fry AM, Schultz SJ, Bartek J, Nigg EA: **Substrate specificity and cell cycle regulation of the Nek2 protein kinase, a potential human homolog of the mitotic regulator NIMA of Aspergillus nidulans.** *J Biol Chem* 1995, **270:**12899-12905.
31. Frantz B, Klatt T, Pang M, Parsons J, Rolando A, Williams H, Tocci MJ, O'Keefe SJ, O'Neill EA: **The activation state of p38 mitogen-activated protein kinase determines the efficiency of ATP competition for pyridinylimidazole inhibitor binding.** *Biochemistry* 1998, **37:**13846-13853.
32. Wu H, Wang ZX: **The mechanism of p21-activated kinase 2 autoactivation.** *J Biol Chem* 2003, **278:**41768-41778.
33. Arias-Romero LE, Chernoff J: **A tale of two Paks.** *Biol Cell* 2008, **100:**97-108.
34. Hill MM, Andjelkovic M, Brazil DP, Ferrari S, Fabbro D, Hemmings BA: **Insulin-stimulated protein kinase B phosphorylation on Ser-473 is independent of its activity and occurs through a staurosporine-insensitive kinase.** *J Biol Chem* 2001, **276:**25643-25646.
35. van der Lugt NM, Domen J, Verhoeven E, Linders K, van der Gulden H, Allen J, Berns A: **Proviral tagging in E mu-myc transgenic mice lacking the Pim-1 proto-oncogene leads to compensatory activation of Pim-2.** *EMBO J* 1995, **14:**2536-2544.
36. Hagiwara M, Brindle P, Harootunian A, Armstrong R, Rivier J, Vale W, Tsien R, Montminy MR: **Coupling of hormonal stimulation and transcription via the cyclic AMP-responsive factor CREB is rate limited by nuclear entry of protein kinase A.** *Mol Cell Biol* 1993, **13:**4852-4859.

37. Chen SJ, Klann E, Gower MC, Powell CM, Sessoms JS, Sweatt JD: **Studies with synthetic peptide substrates derived from the neuronal protein neurogranin reveal structural determinants of potency and selectivity for protein kinase C.** *Biochemistry* 1993, **32:**1032-1039.
38. Golsteyn RM, Mundt KE, Fry AM, Nigg EA: **Cell cycle regulation of the activity and subcellular localization of Plk1, a human protein kinase implicated in mitotic spindle function.** *J Cell Biol* 1995, **129:**1617-1628.
39. Force T, Bonventre JV, Heidecker G, Rapp U, Avruch J, Kyriakis JM: **Enzymatic characteristics of the c-Raf-1 protein kinase.** *Proc Natl Acad Sci U S A* 1994, **91:**1270-1274.
40. Doe C, Bentley R, Behm DJ, Lafferty R, Stavenger R, Jung D, Bamford M, Panchal T, Grygielko E, Wright LL, et al: **Novel Rho kinase inhibitors with anti-inflammatory and vasodilatory activities.** *J Pharmacol Exp Ther* 2007, **320:**89-98.
41. Ross H, Armstrong CG, Cohen P: **A non-radioactive method for the assay of many serine/threonine-specific protein kinases.** *Biochem J* 2002, **366:**977-981.
42. Doi J, Takemori H, Lin XZ, Horike N, Katoh Y, Okamoto M: **Salt-inducible kinase represses cAMP-dependent protein kinase-mediated activation of human cholesterol side chain cleavage cytochrome P450 promoter through the CREB basic leucine zipper domain.** *J Biol Chem* 2002, **277:**15629-15637.
43. Cheng HC, Nishio H, Hatase O, Ralph S, Wang JH: **A synthetic peptide derived from p34cdc2 is a specific and efficient substrate of src-family tyrosine kinases.** *J Biol Chem* 1992, **267:**9248-9256.
44. Hutchison M, Berman KS, Cobb MH: **Isolation of TAO1, a protein kinase that activates MEKs in stress-activated protein kinase cascades.** *J Biol Chem* 1998, **273:**28625-28632.
45. Angeles TS, Yang SX, Steffler C, Dionne CA: **Kinetics of trkA tyrosine kinase activity and inhibition by K-252a.** *Arch Biochem Biophys* 1998, **349:**267-274.
